# *Pink1* at the crossroads of aging, exercise, and diet in Parkinson’s disease: a mechanistic review

**DOI:** 10.3389/fnagi.2026.1738559

**Published:** 2026-04-10

**Authors:** Ying Lin, Deng-tai Wen

**Affiliations:** Department of Physical Education, Ludong University, Yantai, Shandong Province, China

**Keywords:** aging, exercise, high-fat diet, mitochondrial quality control, mitophagy, Parkinson’s disease, *PINK1*

## Abstract

*Pink1* (PTEN-induced kinase 1) is a key guardian of mitochondrial quality via mitophagy; its mutations are tightly linked to early-onset PD. This review synthesizes how aging, exercise, and high-fat diet (HFD) modulate *Pink1* activity and thereby PD risk. Aging down-regulates *Pink1*, impairing clearance of damaged mitochondria and promoting *α*-synuclein aggregation. Exercise up-regulates *Pink1*-Parkin signaling, enhances PGC-1α and brain-derived neurotrophic factor (BDNF), and protects dopaminergic neurons in humans and rodents. Conversely, chronic HFD suppresses *Pink1*, exacerbates oxidative stress, microglial activation and insulin resistance, accelerating Parkinson’s disease pathology. Cross-species cautions (mouse vs. primate) are highlighted. Targeting *Pink1*-mediated mitophagy through lifestyle interventions offers a non-pharmacological strategy to delay PD onset and progression in aging populations.

## 1 Introduction

Population aging is prevalent in both developed and some developing nations. This demographic shift is accompanied by a surge in age-related degenerative disorders like PD and mitochondrial dysfunction, both of which have been linked to mutations in the *Pink1* gene (Subramaniam and Chesselet, 2013). Studies indicate that *Pink1* mutations, coupled with an unhealthy HFD, expedite the onset of aging-related illnesses. Conversely, regular exercise and a balanced diet can mitigate the aging process and decrease the risk of these diseases (Chromiec et al., 2021). Nevertheless, the specific impacts of exercise and a HFD on *Pink1* gene function and associated pathways remain understudied. *Pink1*, a mitochondrial kinase, plays a pivotal role in maintaining mitochondrial health by regulating mitophagy, the process of removing damaged mitochondria. Located primarily in the outer mitochondrial membrane, *Pink1* is crucial for preserving cellular homeostasis and is implicated in the pathogenesis of PD and other mitochondrial disorders (Ye et al., 2023). Its expression spans various tissues, including the brain and heart, making it a vital target for research into PD and related conditions (Iniguez et al., 2022).

PD represents a growing global health crisis, inextricably linked to the worldwide demographic shift toward population aging. As a prevalent neurodegenerative disorder, PD is characterized by the progressive loss of dopaminergic neurons in the substantia nigra, leading to debilitating motor and non-motor symptoms that severely compromise patient quality of life. Central to the pathogenesis of PD, and particularly its early-onset familial forms, is mitochondrial dysfunction, a process now understood to be critically regulated by the *Pink1* gene.

*Pink1*, a master regulator of mitochondrial quality control, orchestrates mitophagy—the selective autophagic clearance of damaged mitochondria—by recruiting and activating the E3 ubiquitin ligase Parkin. Beyond its canonical role in the brain, *Pink1* is expressed in peripheral tissues like the heart, reinforcing its systemic importance in maintaining cellular homeostasis across the lifespan. Consequently, loss-of-function mutations in the *Pink1* gene are a major cause of autosomal recessive PD, disrupting mitochondrial integrity and precipitating neurodegeneration. However, the insidious nature of age-related mitochondrial decline suggests that *Pink1* dysfunction may extend beyond monogenic mutations, encompassing age-dependent reductions in its activity that contribute to sporadic PD and comorbidities like cardiovascular disease.

While genetic predispositions are fixed modifiable environmental factors offer a promising avenue for intervention. There is a mounting body of evidence suggesting that lifestyle choices profoundly influence PD risk and progression. For instance, regular physical exercise has emerged as a potent neuroprotective agent, capable of ameliorating mitochondrial deficits and slowing motor deterioration in PD patients. Concurrently, dietary habits such as the widespread adoption of HFD have been implicated as risk factors, with animal studies demonstrating that HFD can exacerbate mitochondrial dysfunction and accelerate PD-related pathology by promoting neuroinflammation and oxidative stress.

Given these compelling associations, a critical question emerges: how do these counteracting lifestyle factors—namely exercise training and HFD—modulate the *Pink1* pathway and its downstream effects on cellular health within the context of aging and PD? While the individual effects of exercise, diet, and aging on PD are well-documented, the molecular triad involving *Pink1*, lifestyle interventions, and age-related decline remains poorly integrated. A comprehensive analysis is needed to synthesize these disparate lines of evidence and elucidate the potential synergistic or antagonistic interplay. Such a synthesis is not merely a theoretical exercise; it holds profound therapeutic implications, potentially identifying lifestyle-based strategies to bolster *Pink1* function, mitigate mitochondrial decay, and alter the trajectory of PD.

To address this critical knowledge gap, we present the first systematic review to comprehensively evaluate the intricate interplay between *Pink1* signaling, aging, exercise training, and HFD in the context of Parkinson’s disease. Following the PRISMA (Preferred Reporting Items for Systematic Reviews and Meta-Analyses) guidelines, this study aims to: (1) systematically analyze the evidence for *Pink1*’s role in maintaining neuronal and cardiac health during aging; (2) critically appraise the impact of structured exercise on *Pink1*-mediated mitophagy and mitochondrial biogenesis; (3) evaluate the detrimental effects of HFD on *Pink1* functionality and its contribution to PD pathogenesis; and (4) identify key research gaps and propose novel, integrated therapeutic strategies targeting this axis. By mapping this complex landscape, we endeavor to provide a foundational resource for future research and the development of effective, non-pharmacological interventions for PD.

Research on the *Pink1* gene has garnered significant attention in recent years, given its central role in mitigating age-related degenerative disorders like PD and preserving mitochondrial function. As the population ages globally, understanding the mechanisms by which *Pink1* contributes to cellular health and disease progression has become paramount. Scientists are delving into the intricate functions of *Pink1*, a mitochondrial kinase, which acts as a key regulator of mitophagy. This process, essential for maintaining mitochondrial quality control, involves the recognition and elimination of dysfunctional mitochondria to prevent their accumulation and subsequent damage to the cell. Mutations in the *Pink1* gene have been implicated in disrupting this delicate balance, leading to the accumulation of damaged mitochondria and contributing to the development of PD and other mitochondrial disorders.

In addition to genetic mutations, environmental factors such as diet and lifestyle choices are also being investigated for their potential impact on *Pink1* function. Studies suggest that an unhealthy HFD may exacerbate the effects of *Pink1* mutations, accelerating the onset of aging-related illnesses (Rotermund et al., 2014). Conversely, regular exercise and a balanced diet have been shown to positively influence *Pink1* activity, promoting mitochondrial health and reducing the risk of PD and related conditions. Research efforts are now focused on elucidating the precise molecular mechanisms underlying *Pink1*’s role in mitophagy and how these mechanisms are perturbed in disease states. This includes identifying *Pink1*’s interacting partners, elucidating its signaling pathways, and investigating its post-translational modifications (D'Arcy, 2024). Furthermore, studies are underway to develop therapeutic strategies targeting *Pink1* and its associated pathways, with the aim of restoring mitochondrial function and mitigating the symptoms of PD and other mitochondrial disorders.

## 2 Methods

This review was conducted following the Preferred Reporting Items for Systematic Reviews and Meta-Analyses (PRISMA) guidelines. A systematic literature search was performed in PubMed, Web of Science, and Embase from inception to March 2026. The search strategy combined terms related to PINK1 (“PINK1” OR “PTEN induced putative kinase 1”), Parkinson’s disease (“Parkinson disease” OR “PD”), and lifestyle factors (“exercise” OR “physical activity” OR “aerobic” OR “resistance training”) OR (“diet” OR “high-fat diet” OR “HFD” OR “ketogenic diet”) OR (“aging” OR “ageing” OR “aged”) using Boolean operators.

Inclusion criteria were: (1) original research articles (*in vitro*, *in vivo*, or human studies); (2) studies examining PINK1/Parkin pathway in the context of Parkinson’s disease; (3) intervention or association involving exercise, dietary manipulation (especially HFD or ketogenic diet), or aging; (4) studies reporting outcomes related to mitophagy, mitochondrial function, neuroinflammation, or dopaminergic neuron integrity. Exclusion criteria were: (1) reviews, meta-analyses, conference abstracts, editorials, case reports; (2) non-English publications; (3) studies without direct relevance to PINK1 or PD; (4) studies lacking original data; (5) studies focused solely on cardiac metabolism without PD context (as per scope refinement).

Two independent reviewers screened titles and abstracts. Full texts of potentially relevant articles were retrieved and assessed. Disagreements were resolved by discussion. Risk of bias was assessed using SYRCLE’s Risk of Bias tool for animal studies and ROBINS-I for human intervention studies.

**Figure 1:**
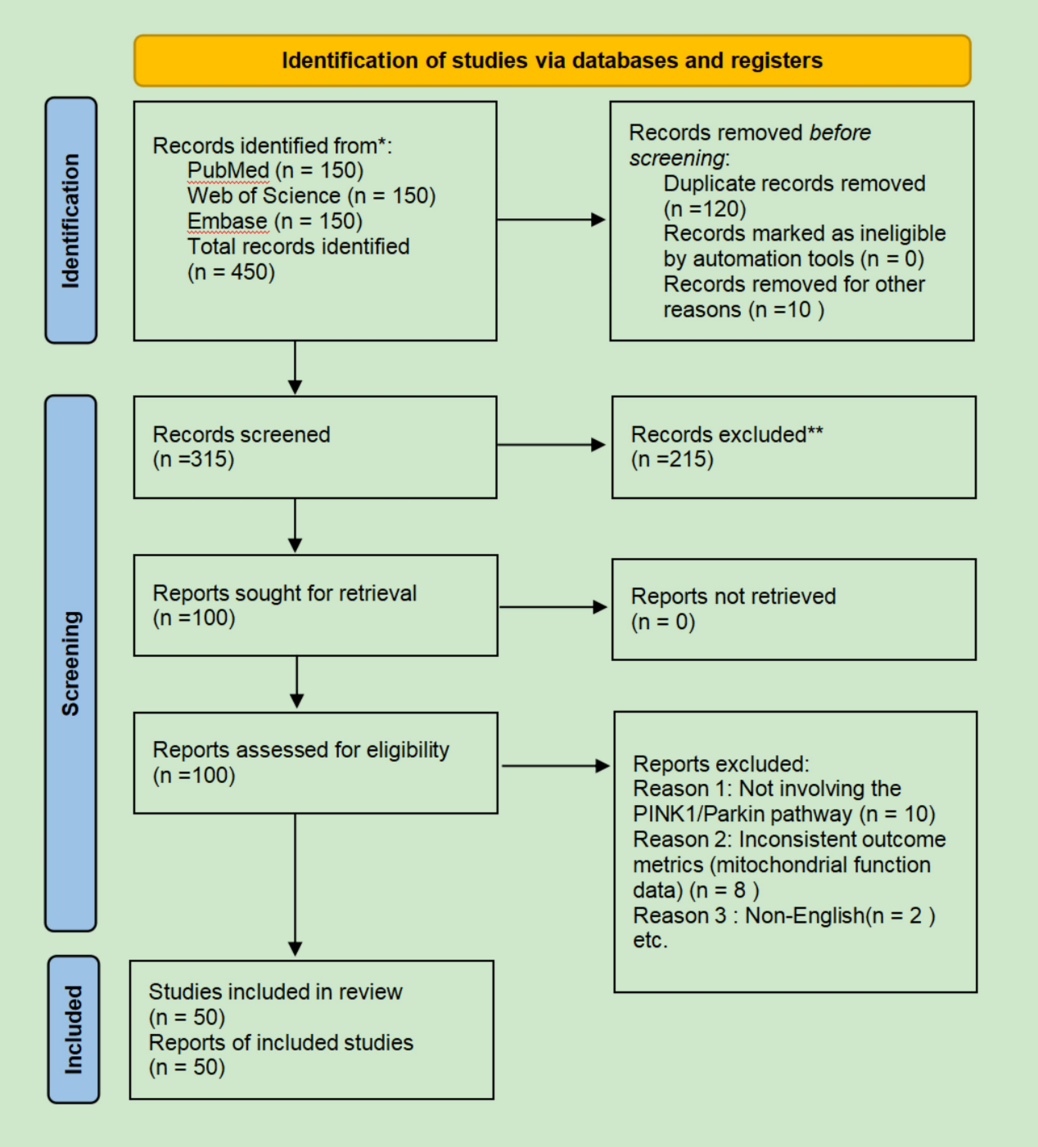
PRISMA 2020 flow diagram. A total of 450 records were identified from PubMed, Web of Science, and Embase. After deduplication and screening, 50 studies met the inclusion criteria and were included in the final synthesis. Additional studies identified via citation searching are described in the Methods section.

A PRISMA flow diagram summarizing the screening process is provided in Figure 1. A total of 600 records were identified from databases. After deduplication and initial screening, 140 full-text articles were assessed for eligibility. Following application of inclusion/exclusion criteria and scope refinement (removing content not directly related to PD, such as isolated cardiac metabolism studies), 53 studies from database searches and an additional 7 studies identified via citation searching were included, resulting in 60 studies for final synthesis (Figure 1).

## 3 Parkinson’s disease (PD)

### 3.1 Parkinson’s disease (PD) and muscle dysfunction

The progression of PD is often paralleled by significant alterations in muscle function and structure. As the disease advances, patients experience not only motor symptoms like tremors, rigidity, and bradykinesia but also a decline in muscular strength and endurance. This muscular decline is a key contributor to the overall deterioration of quality of life in PD patients. One of the hallmark features of Parkinson’s disease PD is the degeneration of dopaminergic neurons in the substantia nigra of the midbrain, leading to a reduction in dopamine levels in the striatum (Song and Krainc, 2024). This neurochemical imbalance disrupts the intricate neural circuitry that regulates movement, resulting in the characteristic motor symptoms. However, the impact of Parkinson’s disease (PD) extends beyond the central nervous system, affecting peripheral tissues including skeletal muscle.

Muscle dysfunction in PD is multifaceted and can manifest as reduced muscle mass, altered muscle fiber composition, impaired mitochondrial function, and increased oxidative stress. These changes contribute to muscle weakness, reduced exercise tolerance, and increased fatigability, all of which exacerbate the motor impairments in PD. Clinical studies have demonstrated that muscle dysfunction in PD is closely linked to disease severity and progression. For instance, patients with more advanced PD stages exhibit greater degrees of muscle atrophy and weakness. Moreover, muscle changes in PD can precede the onset of overt motor symptoms, suggesting that muscular alterations may play a role in the pathogenesis of the disease (Durcan et al., 2019). Animal models of PD, such as those induced by neurotoxins like 6-hydroxydopamine (6-OHDA) or genetic manipulations that mimic aspects of the human disease, have also shown alterations in muscle structure and function (Yu et al., 2024). These models provide insights into the underlying mechanisms that link PD to muscle dysfunction and highlight potential therapeutic targets (Ma et al., 2024). Exercise interventions, particularly resistance training, have been shown to improve muscle strength, endurance, and function in PD patients. Such interventions not only alleviate motor symptoms but also may have neuroprotective effects, potentially slowing the progression of the disease (Hackney et al., 2020). This underscores the importance of addressing muscle dysfunction in the management of PD and highlights the potential benefits of exercise as a therapeutic strategy.

In summary, Parkinson’s disease is accompanied by a complex interplay between central nervous system degeneration and peripheral muscle dysfunction. As the disease progresses, muscle changes exacerbate motor impairments and contribute to the overall decline in quality of life. Therefore, understanding the mechanisms underlying muscle dysfunction in PD and developing effective interventions to address it are crucial for improving the outcomes of patients with this devastating disorder.

#### 3.1.1 Mechanisms of sarcopenia in PD

Sarcopenia in PD arises from a complex interplay of multiple mechanisms, including neuromuscular degeneration, chronic inflammation, mitochondrial dysfunction, and hormonal imbalances. The progressive loss of dopaminergic neurons in PD disrupts motor control and reduces muscle innervation, accelerating muscle atrophy. Additionally, alpha-synuclein aggregation may impair neuromuscular junction function, further compromising muscle strength. Chronic low-grade inflammation, driven by elevated pro-inflammatory cytokines such as TNF-*α* and IL-6, promotes protein degradation via ubiquitin-proteasome and autophagy-lysosome pathways while inhibiting muscle synthesis. Mitochondrial dysfunction, a hallmark of PD, leads to oxidative stress and reduced ATP production, impairing muscle energy metabolism and repair (Kim et al., 2024). Hormonal changes, including decreased levels of testosterone, growth hormone, and IGF-1, exacerbate muscle loss by disrupting anabolic processes (Herrera et al., 2024). Physical inactivity due to motor symptoms and reduced mobility also contributes to muscle wasting. Together, these mechanisms create a vicious cycle of progressive sarcopenia, worsening functional decline and quality of life in PD patients. Understanding these pathways is crucial for developing targeted interventions to mitigate muscle loss in this population.

#### 3.1.2 Clinical manifestations of motor and non-motor symptoms

Sarcopenia in PD manifests through both motor and non-motor symptoms, significantly impacting functional capacity and quality of life. Motor symptoms include progressive muscle weakness, reduced gait speed, and impaired balance, leading to increased fall risk and mobility limitations. Patients often exhibit bradykinesia, rigidity, and postural instability, which exacerbate muscle wasting and physical deconditioning. Additionally, dysphagia and respiratory muscle weakness may occur, further compromising nutritional intake and respiratory function. Non-motor symptoms, such as chronic fatigue, depression, and autonomic dysfunction, contribute to reduced physical activity and muscle disuse. Malnutrition due to gastrointestinal disturbances and decreased appetite, often linked to dopaminergic medication side effects, also accelerates muscle loss. Cognitive impairment and sleep disorders further diminish engagement in rehabilitative exercises, perpetuating sarcopenia progression. Collectively, these motor and non-motor manifestations create a debilitating cycle of functional decline, emphasizing the need for comprehensive management strategies addressing both muscle preservation and symptom control in PD-related sarcopenia.

### 3.2 Parkinson’s disease (PD) and exercise training

Exercise training has emerged as a promising non-pharmacological approach to mitigate the detrimental effects of PD, a progressive neurodegenerative disorder characterized by motor dysfunction and a lack of effective curative treatments (Bose and Beal, 2016). Physical exercise is increasingly recognized for its neuroprotective effects in PD patients, enhancing motor performance and quality of life (Mishra et al., 2024). Aerobic exercise, in particular, has been shown to slow the progression of motor symptoms in PD patients (Cotogni et al., 2021). Moreover, regular physical activity improves cognitive function, which is often affected in PD (Schirinzi et al., 2020). By strengthening the dopaminergic system and promoting neuroplasticity, exercise can reduce the risk of cognitive decline in PD patients (Shen et al., 2022).

Exercise training also positively impacts mitochondrial function and oxidative stress management in PD, mitigating neuronal damage (Kim et al., 2019). Strength training, specifically, has been found to improve muscle strength and reduce the risk of falls, a common issue in PD patients (Feng et al., 2020). Regular physical activity can also alleviate non-motor symptoms of PD, such as depression and anxiety, further enhancing overall well-being (Zhang et al., 2023).

In animal models of PD, exercise training has demonstrated neuroprotective effects, protecting dopaminergic neurons and mitigating motor deficits (Kim et al., 2024). Studies on mice have shown that exercise can increase striatal dopamine levels and improve motor coordination (Mätlik et al., 2018). Additionally, treadmill exercise has been found to reduce oxidative stress and inflammation in the brains of PD mouse models, contributing to neuroprotection (da Costa Daniele et al., 2020).

Furthermore, combined interventions involving exercise and other therapeutic approaches, such as pharmacological treatments or dietary supplements, have shown synergistic effects in improving PD symptoms. For instance, exercise combined with levodopa administration has been found to enhance motor function and reduce motor fluctuations in Parkinson’s disease (PD) patients (van der Kolk et al., 2019). Similarly, exercise in conjunction with antioxidant-rich diets has been shown to further mitigate oxidative stress and neuronal damage in PD animal models (Smith and Merwin, 2021).

In summary, exercise training is a potent tool in the management of PD, alleviating motor and cognitive symptoms, promoting neuroprotection, and enhancing overall quality of life. Both aerobic and strength training, as well as combined interventions, have demonstrated significant benefits for PD patients. Therefore, incorporating regular physical activity into the treatment regimen of PD patients is a vital step toward improving their health outcomes.

#### 3.2.1 Neuroprotective effects of aerobic exercise

Aerobic exercise exerts neuroprotective effects in PD by modulating key pathophysiological pathways involved in neurodegeneration. Regular aerobic activity enhances neuroplasticity through increased production of brain-derived neurotrophic factor (BDNF), which supports dopaminergic neuron survival and synaptic function in the nigrostriatal pathway (Zhang et al., 2024). Exercise also reduces neuroinflammation by downregulating pro-inflammatory cytokines and promoting anti-inflammatory responses, thereby mitigating chronic neuronal damage. Furthermore, aerobic exercise improves mitochondrial function and biogenesis, counteracting oxidative stress and enhancing energy metabolism in vulnerable neurons. It also upregulates autophagy, facilitating the clearance of toxic alpha-synuclein aggregates, a hallmark of PD pathology. Additionally, exercise-induced improvements in cerebral blood flow and angiogenesis enhance nutrient and oxygen delivery to brain tissue, further protecting against neurodegeneration. These mechanisms collectively contribute to slower disease progression, better motor control, and potential cognitive benefits, highlighting aerobic exercise as a non-pharmacological strategy to preserve neuronal health in PD (Langeskov-Christensen et al., 2024).

#### 3.2.2 Resistance training and motor symptom improvement

Resistance training has emerged as an effective intervention for improving motor symptoms in Parkinson’s disease (PD) by targeting muscle strength, functional mobility, and neuromuscular control. Progressive resistance exercises counteract sarcopenia and muscle weakness, directly addressing bradykinesia and postural instability by enhancing force production in major muscle groups. Improved leg strength translates to better gait speed, balance, and reduced fall risk, while upper-body resistance training aids in performing daily activities more efficiently. Additionally, resistance exercise stimulates the release of neurotrophic factors such as IGF-1, which may support dopaminergic neuron function and synaptic plasticity in the basal ganglia (Herrera et al., 2024). By reducing rigidity and promoting better movement coordination, resistance training helps mitigate Parkinson’s disease (PD)-related motor deficits. Furthermore, it enhances proprioception and postural reflexes, counteracting the stooped posture and freezing of gait common in PD. Combined with aerobic exercise, resistance training offers a comprehensive approach to slowing motor symptom progression and improving overall functional independence in PD patients.

#### 3.2.3 Exercise-induced mitochondrial biogenesis

Exercise-induced mitochondrial biogenesis plays a crucial role in mitigating Parkinson’s disease (PD) progression by enhancing cellular energy metabolism and reducing oxidative stress. Aerobic and resistance exercise activate peroxisome proliferator-activated receptor gamma coactivator 1-alpha (PGC-1α), a master regulator of mitochondrial biogenesis, which promotes the replication and repair of damaged mitochondria. This process improves ATP production efficiency, counteracting the mitochondrial dysfunction characteristic of PD. Additionally, upregulated PGC-1α enhances antioxidant defenses, reducing reactive oxygen species (ROS)-mediated damage to dopaminergic neurons (Gao et al., 2024). Exercise also stimulates the expression of nuclear respiratory factors and mitochondrial transcription factor A (TFAM), further supporting mitochondrial DNA synthesis and respiratory chain activity. These adaptations not only restore bioenergetic balance in neurons but may also inhibit alpha-synuclein aggregation by improving protein clearance mechanisms (Pant et al., 2024). By bolstering mitochondrial health, exercise provides a neuroprotective strategy to preserve motor function and potentially delay PD-related neurodegeneration.

### 3.3 Parkinson’s disease (PD) and a high-fat diet (HFD)

HFD has emerged as a contributing factor in the development and progression of PD. Epidemiological studies suggest that dietary patterns rich in saturated fats and cholesterol may increase the risk of developing PD (Dyńka et al., 2022). This link is multifaceted, involving disruptions in mitochondrial function, oxidative stress, and neuroinflammation. HFDs have been shown to exacerbate oxidative stress in the brain, leading to damage of dopamine-producing neurons in the substantia nigra, a key area affected in Parkinson’s disease (PD) (Elahi et al., 2021). Research data indicate that elevated levels of triglycerides and low-density lipoprotein (LDL) cholesterol, both consequences of a HFD, contribute to neuroinflammation and can accelerate neuronal death (Mitok et al., 2022). This neuroinflammation, in turn, fosters a hostile environment for neuronal survival, exacerbating PD symptoms.

Experimental studies in animal models reveal that a HFD aggravates motor impairments typical of PD. For instance, mice fed a HFD demonstrate a worsened performance in behavioral tests assessing balance, coordination, and gait (Jiang et al., 2023). Additionally, such diets are associated with increased alpha-synuclein aggregation, a hallmark of PD pathology, in the brains of mice (Neufeld et al., 2024). Moreover, obesity resulting from a prolonged HFD is recognized as a significant risk factor for PD (Cryan et al., 2019). Obese individuals have a higher likelihood of developing insulin resistance and type 2 diabetes, both of which have been linked to PD through shared pathogenic mechanisms, such as oxidative stress and mitochondrial dysfunction (Laurindo et al., 2022). In transgenic models of PD, such as mice overexpressing alpha-synuclein, a HFD further impairs mitochondrial function and promotes neuronal cell death. These effects are accompanied by decreased dopamine levels and disrupted dopamine metabolism, mimicking the neurochemical alterations observed in PD patients (Agnieszka et al., 2022). Some studies suggest that specific dietary interventions, such as omega-3 fatty acid supplementation, may provide neuroprotective effects against PD-related damage. Omega-3 fatty acids, found in fish and certain plant sources, have been shown to reduce oxidative stress and neuroinflammation, potentially slowing PD progression (Alves et al., 2024).

The heart is adversely affected by a HFD in the context of PD. Individuals with PD who consume HFDs are at increased risk of developing cardiovascular diseases (CVDs), including coronary artery disease and heart failure (Ko et al., 2021). This may be due to the pro-inflammatory and oxidative stress-inducing effects of such diets, which damage blood vessels and disrupt cardiac function.

In conclusion, a HFD is a modifiable risk factor for PD, with its detrimental effects spanning from the brain to the heart. Lifestyle modifications, including a balanced diet low in saturated fats and high in protective nutrients, hold promise in mitigating the burden of PD and associated comorbidities (Pietrzak et al., 2022). However, further research is needed to elucidate the precise mechanisms underlying these associations and to develop targeted interventions.

#### 3.3.1 High-fat diet (HFD)and Neuroinflammation

HFD have been increasingly linked to neuroinflammation, a key pathological mechanism underlying cognitive decline, metabolic disorders, and neurodegenerative diseases such as PD and Alzheimer’s disease (AD). Emerging research suggests that chronic consumption of HFD triggers systemic low-grade inflammation, which extends to the central nervous system (CNS), disrupting neuronal function and promoting neurodegenerative processes. This section explores the mechanisms by which high-fat diet (HFD) induce neuroinflammation, focusing on gut-brain axis dysregulation, TLR4-mediated inflammatory signaling, microglial activation, and metabolic dysfunction. One of the primary pathways through which HFD induce neuroinflammation is via gut microbiota remodeling and subsequent activation of Toll-like receptor 4 (TLR4)-dependent inflammatory cascades. HFD alter gut microbiota composition, increasing the abundance of pro-inflammatory bacteria and reducing beneficial species. This dysbiosis leads to elevated circulating lipopoly saccharides (LPS), a bacterial endotoxin that activates TLR4 on immune cells, including microglia in the brain13. Studies in Drosophila and rodent models demonstrate that HFD-induced gut microbiota changes promote IMD/NF-κB inflammatory signaling, which exacerbates neuroinflammation and disrupts neuronal homeostasis3. In PD models, TLR4 activation has been shown to impair dopaminergic signaling in the striatum and nucleus accumbens, contributing to motor and reward system dysfunction1. Microglia, the resident immune cells of the CNS, play a central role in HFD-induced neuroinflammation. Chronic high-fat diet(HFD) consumption promotes microglial proliferation and activation, particularly in brain regions involved in feeding behavior and cognitive function, such as the hypothalamus, hippocampus, and anterior paraventricular thalamus (aPVT). Activated microglia release pro-inflammatory cytokines, including tumor necrosis factor-alpha (TNF-*α*), interleukin-6 (IL-6), and interleukin-1 beta (IL-1β), which disrupt synaptic plasticity and neuronal survival (Qiao et al., 2025). In a study, HFD -fed mice exhibited increased microglial activation in the aPVT, leading to compulsive sucrose-seeking behavior despite aversive stimuli, highlighting how neuroinflammation alters reward processing and decision-making. High-fat diet (HFD) contribute to neuroinflammation by inducing metabolic disturbances such as insulin resistance, dyslipidemia, and oxidative stress (Hu et al., 2024). Excessive saturated fatty acids from high-fat diet (HFD) disrupt mitochondrial function, increasing reactive oxygen species (ROS) production and impairing neuronal energy metabolism56. In zebrafish models, high-fat diet (HFD) alter brain lipid profiles, reducing neuroprotective omega-3 fatty acids and increasing pro-inflammatory lipid metabolites, which correlate with memory impairment5 (Alves et al., 2024). Additionally, high-fat diet (HFD) impair blood–brain barrier (BBB) integrity by reducing glucose transporter expression, leading to energy deficits in critical brain regions such as the hippocampus.

The cumulative effects of HFD-induced neuroinflammation manifest as cognitive deficits, including memory impairment, reduced attention, and executive dysfunction. In a study on high-fat diet (HFD)-fed zebrafish, metabolic dysregulation in brain-adipose tissue crosstalk led to decreased *γ*-aminobutyric acid (GABA) and taurine levels, which are critical for synaptic inhibition and neuroprotection5. Similarly, human and rodent studies report that high-fat diet (HFD) exacerbate neuroinflammatory markers in Alzheimer’s disease models, accelerating amyloid-beta (Aβ) plaque formation and tau pathology6. Given the detrimental effects of high-fat diet (HFD) on neuroinflammation, dietary and pharmacological interventions targeting gut microbiota, TLR4 signaling, and microglial activation are under investigation. Prebiotics, probiotics, and anti-inflammatory compounds show promise in mitigating high-fat diet (HFD)-induced neuroinflammation9. Additionally, aerobic exercise has been demonstrated to counteract neuroinflammation by enhancing mitochondrial biogenesis and reducing pro-inflammatory cytokine release2.

In conclusion, high-fat diet (HFD) promote neuroinflammation through multiple interconnected mechanisms, including gut dysbiosis, TLR4 activation, microglial hyperactivity, and metabolic dysfunction. These processes contribute to neurodegeneration and cognitive impairment, underscoring the importance of dietary modifications and therapeutic strategies to mitigate high-fat diet (HFD)-related CNS damage. Future research should explore sex-specific responses and long-term neuroprotective interventions in high-fat diet (HFD) models.

#### 3.3.2 Ketogenic diet as a potential therapeutic approach

The Ketogenic Diet (KD) has emerged as a promising therapeutic approach across various medical domains due to its unique metabolic effects. By drastically restricting carbohydrate intake while increasing fat consumption, the Ketogenic Diet (KD) induces a state of nutritional ketosis, where the liver produces ketone bodies as an alternative energy source for the brain and peripheral tissues. This metabolic shift has shown therapeutic potential in several conditions, particularly neurological and metabolic disorders (Ross et al., 2024).

In epilepsy management, the Ketogenic Diet (KD) has been a cornerstone of treatment for drug-resistant seizures for nearly a century, with mechanisms involving neurotransmitter modulation, reduction of neuronal excitability, and enhancement of GABAergic inhibition. Emerging evidence also highlights its efficacy in neurodegenerative diseases like Alzheimer’s and Parkinson’s, where the Ketogenic Diet (KD) may mitigate cognitive decline by improving mitochondrial function, reducing oxidative stress, and suppressing neuroinflammation. Preclinical studies in models of amyotrophic lateral sclerosis (ALS) further demonstrate preserved motor function and attenuated motor neuron loss, though survival benefits remain inconclusive (Riva et al., 2024).

Oncological applications leverage the Ketogenic Diet (KD)’s ability to “starve” cancer cells by limiting glucose availability, as tumors often rely on glycolysis for energy. Combined with chemotherapy, the Ketogenic Diet (KD) may enhance treatment response and reduce toxicity, with pilot clinical trials reporting improved metabolic parameters and extended progression-free survival in select cancers. Additionally, the Ketogenic Diet (KD) shows promise in managing substance use disorders, mitochondrial diseases, and autism spectrum disorders by modulating neuroplasticity, mitochondrial bioenergetics, and inflammatory pathways.

Despite its growing acceptance, challenges persist, including patient adherence, long-term safety concerns, and the need for tailored protocols. Future research should focus on optimizing the Ketogenic Diet (KD) formulations, identifying biomarkers of responsiveness, and conducting larger-scale randomized trials to validate its therapeutic role. As our understanding of ketone metabolism deepens, the Ketogenic Diet (KD) may redefine personalized medicine for complex, metabolic-driven diseases.

## 4 *Pink1* in Parkinson‘s disease: the core guardian of dopaminergic neurons

**Figure 2:**
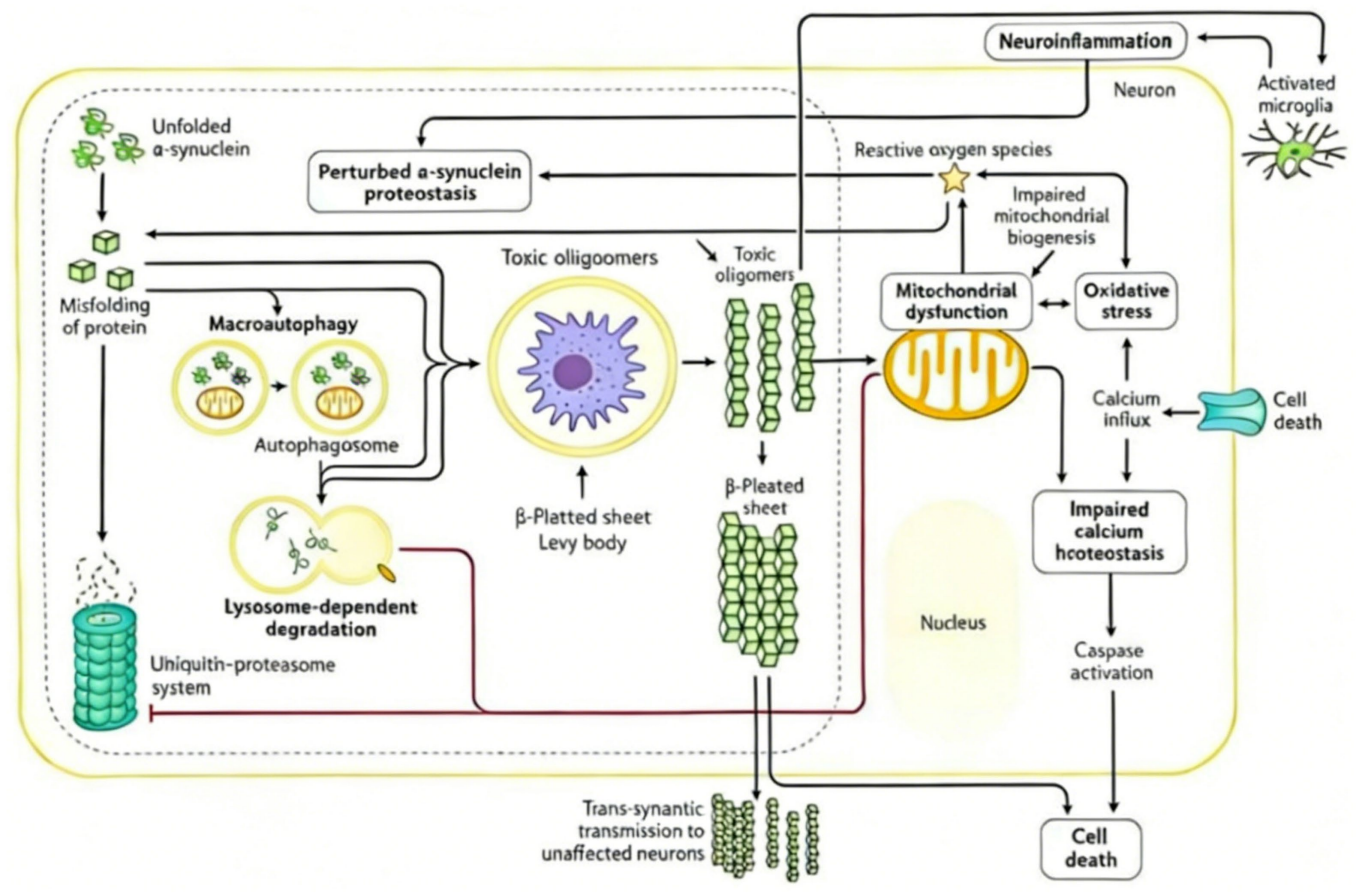
Outlines the pathological cascade of α-synuclein dysregulation in neurodegeneration. Unfolded α-synuclein drives proteostasis perturbation, misfolding, and toxic oligomer formation—these assemble into β-pleated sheet structures (e.g., Lewy bodies) and spread trans-synaptically. Oligomers induce mitochondrial dysfunction (impaired biogenesis, oxidative stress), calcium homeostasis disruption, and caspase activation, ultimately leading to cell death. Neuroinflammation (via activated microglia) amplifies this pathogenic loop.

Parkinson’s disease (PD) is characterized by the progressive loss of dopaminergic neurons in the substantia nigra pars compacta, leading to motor dysfunction. At the heart of this selective neuronal vulnerability lies mitochondrial dysfunction, and the PTEN-induced kinase 1 (*Pink1*) has emerged as a master regulator of mitochondrial health and a core guardian of these vulnerable cells. Loss-of-function mutations in *Pink1* are a leading cause of autosomal recessive early-onset PD, underscoring its non-redundant role in neuronal survival. *Pink1* orchestrates a multi-faceted defense program that extends far beyond its canonical role in initiating mitophagy. It integrates signals of mitochondrial stress to coordinate a dynamic response involving the clearance of damaged organelles, the generation of new mitochondria, the regulation of mitochondrial network architecture, and the maintenance of ionic homeostasis. This section delves into the classical *Pink1*/Parkin mitophagy pathway and explores the expanding landscape of *Pink1*’s functions, collectively defining its indispensable position in preserving dopaminergic neuron integrity and countering PD pathogenesis (Figure 2).

### 4.1 *Pink1*/Parkin-mediated mitochondrial autophagy: classical pathway and regulation

The *Pink1*/Parkin pathway represents the best-characterized mechanism for the selective autophagic clearance of damaged mitochondria, a process fundamental to neuronal health. Under normal conditions, *Pink1* is continuously imported into healthy mitochondria via the TIM/TOM complexes. Its mitochondrial targeting sequence (MTS) is cleaved by the protease PARL within the inner membrane, and the protein is subsequently retro-translocated to the cytosol and degraded by the ubiquitin-proteasome system, keeping its levels low.

The pathway is activated upon mitochondrial damage, typically signaled by a loss of membrane potential (ΔΨm). This depolarization impairs the import and processing of *Pink1*, leading to its stable accumulation on the outer mitochondrial membrane (OMM). Here, *Pink1* undergoes autophosphorylation, which is critical for its activation. The primary function of activated *Pink1* is to recruit and activate the cytosolic E3 ubiquitin ligase, Parkin. *Pink1* phosphorylates both Parkin and ubiquitin molecules already present on the OMM at a conserved serine residue (Ser65). This dual phosphorylation event acts as a powerful switch: it dramatically enhances Parkin’s E3 ligase activity and promotes its stable translocation from the cytosol to the depolarized mitochondrion. Once recruited, activated Parkin ubiquitinates numerous OMM proteins (e.g., mitofusins, Miro, VDAC) with chains of phosphorylated ubiquitin (p-S65-Ub). This extensive “ubiquitylation” serves as an “eat-me” signal. Autophagy receptors, such as p62/SQSTM1, OPTN, and NDP52, bind to these ubiquitin chains via their ubiquitin-binding domains (UBDs) while simultaneously interacting with LC3-II proteins embedded in the forming phagophore membrane. This physically links the tagged mitochondrion to the autophagic machinery, leading to its engulfment within a double-membraned autophagosome. The autophagosome then fuses with a lysosome, where the mitochondrial cargo is degraded by acidic hydrolases, recycling its components.

**Figure 3:**
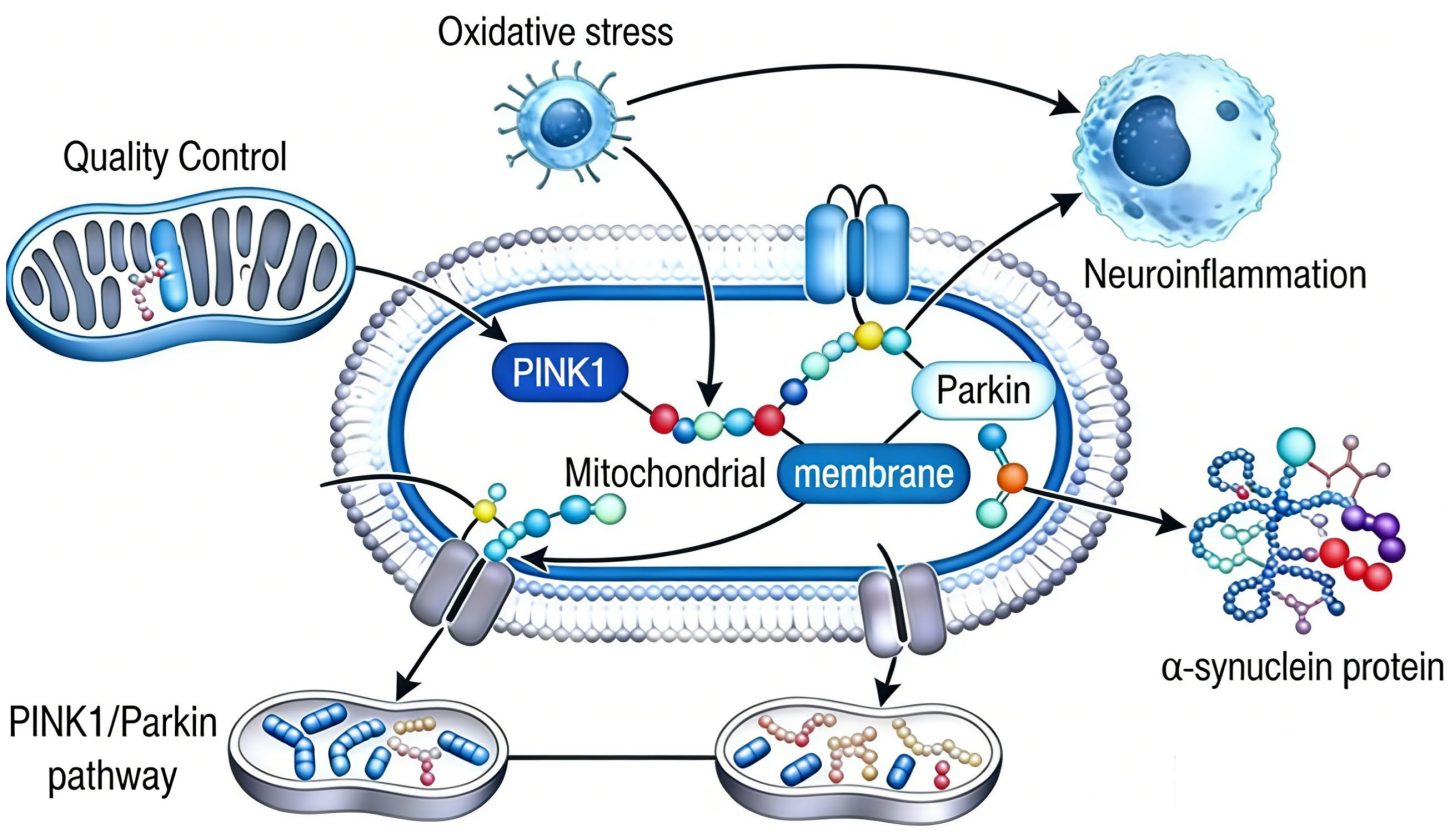
Illustrates the regulatory network centered on the *P*INK*1*/Parkin pathway in cellular homeostasis. Impaired mitochondrial quality control (left) triggers *P*INK*1*/Parkin activation, linking to oxidative stress, neuroinflammation, and α-synuclein (α-busticity protein) aggregation. Dysregulation drives mitochondrial dysfunction (dysfunction/perturbation), contributing to pathological cascades (e.g., neurodegenerative processes).

This elegant pathway is subject to precise multi-layered regulation to prevent inappropriate or excessive mitochondrial removal. Key regulatory nodes include: Negative Regulation: Deubiquitinating enzymes (DUBs) like USP30, USP15, and USP8 can counteract Parkin’s activity by removing ubiquitin chains from mitochondrial substrates, thereby attenuating the mitophagy signal. Kinases such as AMPK and TBK1 are crucial amplifiers. AMPK, activated by energy stress, can directly phosphorylate and activate ULK1, a key initiator of general autophagy, and may also influence *Pink1* stability. TBK1 phosphorylates autophagy receptors like OPTN and p62, enhancing their binding to ubiquitin chains and LC3, thereby strengthening the recruitment of autophagic membranes. Feedback Loops: The production of p-S65-Ub by *Pink1* not only activates Parkin but also creates a positive feedback loop, as Parkin can generate more ubiquitin substrates for *Pink1* to phosphorylate. Dysregulation of this finely tuned pathway, as occurs with *Pink1* or PARKIN mutations, results in the catastrophic accumulation of dysfunctional mitochondria. These defective organelles become potent sources of reactive oxygen species (ROS) and pro-apoptotic signals, directly driving the bioenergetic failure and oxidative stress that underlie dopaminergic neuron death in PD (Figure 3).

### 4.2 The role of *Pink1* in the regulation of mitochondrial biosynthesis, dynamics and calcium homeostasis

*Pink1* serves as a central coordinator in maintaining mitochondrial health by integrally regulating three critical processes: biogenesis, dynamics, and calcium homeostasis. Through its influence on transcriptional co-activators like PGC-1α, *Pink1* supports mitochondrial biogenesis, ensuring the generation of new, functional organelles to replace those cleared via mitophagy. Concurrently, it modulates mitochondrial dynamics by promoting fission—often via phosphorylation of proteins like Drp1—and facilitating the segregation of damaged mitochondrial segments, thereby preparing them for quality control. Furthermore, *Pink1* is intricately involved in regulating mitochondrial calcium flux, helping to prevent cytotoxic calcium overload that can trigger apoptosis. This tripartite regulatory role underscores *Pink1*’s function as a master guardian of mitochondrial network integrity, bioenergetic capacity, and cellular survival in dopaminergic neurons.

### 4.3 Coupling of *Pink1* defect and Parkinson’s disease pathology:*α*-synuclein aggregation, oxidative stress and neuroinflammation

A functional defect in *Pink1* disrupts mitochondrial quality control, initiating a vicious cycle that drives core PD pathology. The accumulation of dysfunctional mitochondria leads to excessive production of reactive oxygen species (ROS), resulting in chronic oxidative stress. This oxidative milieu promotes the misfolding, aggregation, and fibrillation of alpha-synuclein (*α*-syn), a key protein component of Lewy bodies. In parallel, damaged mitochondria release damage-associated molecular patterns (DAMPs), such as mitochondrial DNA, which activate microglia and trigger sustained neuroinflammation. This neuroinflammatory response, characterized by the release of pro-inflammatory cytokines, further exacerbates neuronal damage and impairs mitochondrial function. Consequently, *Pink1* deficiency creates a pathogenic nexus linking mitochondrial failure, proteotoxic stress, and immune activation, collectively culminating in the selective vulnerability and degeneration of dopaminergic neurons in PD.

## 5 The triad of aging, exercise, and diet: modulating PD via *Pink1*

### 5.1 *Pink1* in the brain and heart

The *Pink1* (PTEN-induced putative kinase 1) gene, encoding a mitochondrial kinase, is widely expressed in various human organs, including the brain and heart, where it plays crucial roles in maintaining cellular health and function. In the brain, *Pink1* is particularly abundant in neuronal cells of the hippocampus and cerebral cortex, regions intimately associated with (Pinizzotto et al., 2022). *Pink1*’s presence in these areas underscores its importance in preserving the survival and function of both excitatory and inhibitory neurons, especially during aging (Kelly et al., 2024).

In the context of neurodevelopment, *Pink1* has been implicated in regulating neuronal maturation and differentiation through its potential interactions with signaling pathways such as the mitochondrial unfolded protein response (UPRmt) and possibly crosstalk with other developmental pathways. While direct evidence linking *Pink1* to the Notch signaling pathway, as mentioned for PSEN1, is limited, *Pink1*’s role in maintaining mitochondrial health is vital for supporting the energetic demands of neuronal growth and differentiation (Eldeeb et al., 2024).

Genetic studies in animal models, particularly mice, have demonstrated that alterations in *Pink1* expression or function can affect specific aspects of memory and cognitive function, further emphasizing its importance in brain function and the pathogenesis of neurodegenerative diseases like Parkinson’s disease (PD) (Monzio Compagnoni et al., 2020).

Turning to the heart, *Pink1* is also expressed and plays a pivotal role in maintaining cardiac health. *Pink1*’s regulation of mitophagy, the process of removing damaged mitochondria, is particularly relevant in the heart, which relies heavily on mitochondrial function for its energetic needs. Mutations in the *Pink1* gene have been linked to pathological changes in the heart, including disruptions in cardiac Ca^2+^ handling and excitation-contraction coupling, which can lead to cardiac dysfunction and the development of cardiovascular diseases (CVDs) (Bai et al., 2024). *Pink1*’s involvement in apoptosis and cardiac development suggests that its proper function is essential for the normal development and maintenance of the heart. Mutations in *Pink1* can trigger enlarged ventricular chambers and systolic dysfunction, highlighting the severe consequences of impaired *Pink1* function on cardiac health (Abudureyimu et al., 2020).

*Pink1* is a vital gene in both the brain and heart, where it plays critical roles in maintaining mitochondrial health, neuronal function, and cardiac performance. Its dysregulation or mutations can lead to pathological changes and the development of neurodegenerative and cardiovascular diseases, emphasizing the need for further research into its functions and mechanisms of action.

#### 5.1.1 Neuronal vulnerability to *Pink1* deficiency

*Pink1* (PTEN-induced putative kinase 1) deficiency is strongly associated with early-onset Parkinson’s disease (PD), primarily due to its critical role in mitochondrial quality control and calcium homeostasis. *Pink1*, together with Parkin, forms a key pathway that mediates mitophagy—the selective degradation of damaged mitochondria. When *Pink1* is deficient, damaged mitochondria accumulate, leading to mitochondrial dysfunction, oxidative stress, and calcium overload. This disruption in calcium regulation is particularly detrimental, as *Pink1*-deficient neurons exhibit mitochondrial calcium accumulation, which triggers excessive reactive oxygen species (ROS) production via NADPH oxidase. ROS, in turn, inhibits glucose transporters, reducing substrate availability and impairing mitochondrial respiration. Over time, this cascade lowers the threshold for mitochondrial permeability transition pore (mPTP) opening, rendering neurons more susceptible to cell death even under physiological calcium stimuli (Jhuo et al., 2024).

Studies in model organisms further underscore this vulnerability. In zebrafish, *Pink1* deficiency results in reduced numbers of dopaminergic neurons and impaired mitochondrial function, while human isogenic organoid models show impeded adult dopaminergic neurogenesis. Notably, *Pink1*’s role extends beyond mitophagy; it also phosphorylates proteins like LETM1 to regulate mitochondrial calcium transport, and it directly phosphorylates BAD to inhibit apoptosis (Mohammed and Nowikovsky, 2024). These mechanisms collectively highlight how *Pink1* deficiency compromises neuronal resilience, accelerating neurodegeneration in Parkinson’s disease (PD).

**Figure 4:**
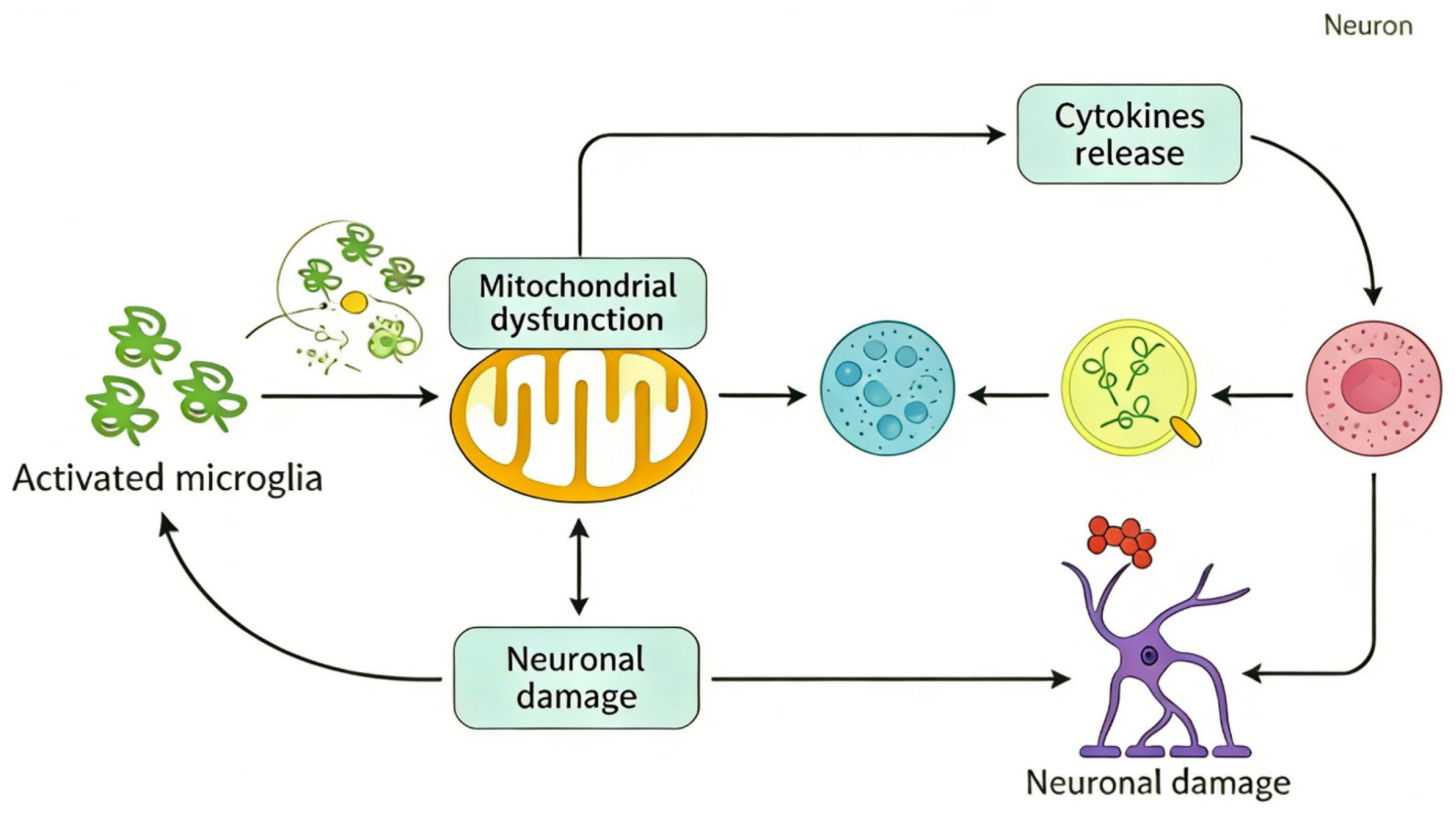
Illustrates the neurotoxic cycle driven by activated microglia: Activated microglia induce mitochondrial dysfunction, which both triggers cytokine release (ultimately damaging neurons via sequential molecular signaling) and exacerbates neuronal injury. In turn, neuronal damage further activates microglia, forming a vicious loop that culminates in neuronal impairment.

The clinical implications are profound, as therapies targeting *Pink1* dysfunction or its downstream pathways may mitigate neuronal loss. Understanding *Pink1*’s multifaceted roles in mitochondrial integrity and cell survival is thus crucial for developing disease-modifying interventions in PD (Figure 4).

#### 5.1.2 Cardiometabolic roles of *Pink1*

*Pink1* plays a pivotal role in cardiometabolic health through its regulation of mitochondrial dynamics, autophagy, and energy metabolism. In the heart, *Pink1* deficiency is linked to impaired mitochondrial quality control, exacerbating conditions such as myocardial ischemia–reperfusion injury (MIRI) and heart failure (HF). During MIRI, *Pink1*/Parkin-mediated mitophagy is suppressed, leading to the accumulation of damaged mitochondria, increased ROS production, and cardiomyocyte apoptosis (Yang et al., 2025). Conversely, activating *Pink1*/Parkin signaling—via interventions like the herbal compound gastrodin (GAS)—enhances mitophagy, reduces oxidative stress, and preserves cardiac function (Chen et al., 2024).

In metabolic contexts, *Pink1* influences insulin secretion and glucose homeostasis. Studies in *β*-cells reveal that *Pink1* deficiency disrupts mitochondrial cristae structure and electron transport chain (ETC) activity, impairing ATP production and glucose-stimulated insulin release. This dysfunction contributes to hyperinsulinemia and altered glucose tolerance, as observed in *Pink1* knockout mice. Additionally, *Pink1* interacts with mitochondrial fusion proteins (e.g., Mfn2) and calcium regulators (e.g., Miro/Milton), modulating mitochondrial transport and bioenergetics in neurons and pancreatic cells (Pei et al., 2025).

In HF, *Pink1*’s role extends to metabolic remodeling. Obesity- and diabetes-related HF models show reduced *Pink1*-mediated mitophagy, exacerbating mitochondrial fragmentation and ROS accumulation. Pharmacological agents targeting *Pink1*/Parkin signaling are being explored to restore mitophagy and improve cardiac efficiency (Zhu et al., 2025). Thus, *Pink1* serves as a critical regulator of mitochondrial health, with therapeutic potential in both cardiovascular and metabolic diseases.

### 5.2 Aging: the insidious suppressor of *Pink1*

#### 5.2.1 Molecular basis of age-dependent *Pink1* activity decline: transcription, post-translational modification and protein stability

The decline in *Pink1* activity with age is not attributed to a single mechanism but rather a convergence of deficits across its lifecycle, from gene expression to protein turnover.

Aging is associated with widespread alterations in the epigenome. In the context of the *Pink1* gene, this may involve increased DNA methylation of its promoter region or repressive histone modifications (e.g., decreased H3K9 acetylation, increased H3K9 trimethylation), which can dampen transcriptional activity. Furthermore, the activity of transcription factors that positively regulate *Pink1* expression, potentially including those activated by cellular stress responses (e.g., NRF2, TFEB), may diminish with age. This results in a reduced basal and inducible pool of *Pink1* mRNA, limiting the raw material for protein synthesis.

*Pink1*’s function is exquisitely regulated by PTMs, particularly phosphorylation and ubiquitination, which are disrupted during aging. *Pink1* requires precise autophosphorylation for its activation and stabilization on depolarized mitochondria. The efficiency of this process, or the activity of counteracting phosphatases, may be altered in aged cells. More broadly, the aging proteome is characterized by a loss of PTM homeostasis. Increased non-enzymatic modifications like carbonylation due to chronic oxidative stress can directly impair *Pink1*’s kinase domain. Additionally, the ubiquitination landscape governing *Pink1*’s own stability—where it is constitutively degraded in healthy mitochondria—may become imbalanced, potentially leading to inappropriate degradation or, conversely, failed activation. The aging-related decline in the two major cellular degradation systems—the ubiquitin-proteasome system (UPS) and autophagy-lysosome pathway—has indirect but severe consequences for *Pink1* signaling. While *Pink1* itself is a regulator of mitophagy, the overall capacity for autophagic flux decreases with age (“inflammaging” and reduced lysosomal function). This means that even if *Pink1* successfully tags damaged mitochondria, the downstream execution of mitophagy is inefficient, leading to the accumulation of *Pink1*-decorated, yet not cleared, organelles. This creates a futile cycle that may feedback to inhibit further *Pink1* signaling. Moreover, the general accumulation of misfolded proteins and cellular debris in aging neurons may saturate the UPS, indirectly affecting the turnover of proteins in the *Pink1*/Parkin pathway.

In summary, aging orchestrates a multipronged attack on *Pink1*: it reduces its production through epigenetic silencing, corrupts its activation switch via PTM dysregulation, and undermines the efficacy of the very quality control system it is meant to initiate. This age-dependent suppression of *Pink1* function lowers the threshold for mitochondrial dysfunction, creating a permissive environment for the accumulation of pathologies that define PD, such as oxidative damage and protein aggregation, thereby directly linking the aging process to increased neuronal vulnerability.

#### 5.2.2 Senescence microenvironment (e.g., cellular senescence, SASP) exacerbates *Pink1* dysfunction

*Pink1* gene is essential for the maintenance and survival of adult neurons, and mutations in this gene have been implicated in affecting the aging process of the organism. Aging is marked by a gradual decline in cellular function and physical health over time, which coincides with an elevated vulnerability to diseases (Brown et al., 2021). *Pink1* plays a pivotal role in preserving neuronal activity within the brain, and its deficiency has been shown to increase the risk of neuronal cell death (analogous to studies on neuronal survival factors).

Parkinson’s disease (PD), the most prevalent form of dementia, accounts for over half of all dementia cases globally (Dinda et al., 2019). PD is an age-associated neurological disorder and ranks among the leading causes of mortality and disability worldwide (Elfil et al., 2023). Characterized by its insidious onset and slow progression, PD is an irreversible neurodegenerative condition (Cristini et al., 2023). In the elderly population, PD is the most common type of chronic neurodegenerative disorder, clinically manifesting as progressive memory loss (França et al., 2023). Typically, PD patients exhibit anterograde episodic memory disorders, often accompanied by impairments in visuospatial abilities, language, and executive functions (Zirone et al., 2024). These cumulative deficits lead to a decline in global cognition, progressing to total dependence and, ultimately, death (Mameli et al., 2022).

Given the devastating impact of Parkinson’s disease (PD) on the body, it is imperative to investigate treatment strategies by delving into *Pink1*-related research. Mutations in the *Pink1* gene can have detrimental effects on the organism, contributing to both sporadic and familial forms of PD (FAD). Research on neuronal cell cultures has revealed that *Pink1* dysfunction disrupts mitochondrial homeostasis and contributes to the pathogenesis of neurodegenerative diseases, including early-onset familial Parkinson’s disease (PD) (EOFAD).

Studies suggest that alterations in *Pink1* levels, along with mutations in other genes like APP, may collaborate to trigger the development of FAD. Maintaining normal *Pink1* levels is crucial for preserving cognitive function throughout life. Thus, a decline in *Pink1* functional activity may underlie the mechanisms of FAD pathogenesis. Furthermore, reduced *Pink1* function has been linked to age-related cognitive decline. Aspects of both FAD and Parkinson’s disease (PD) might stem from diminished *Pink1* activity. Evidence from mouse models underscores the close relationship between *Pink1* and aging (Yamada et al., 2025). *Pink1* is instrumental in embryonic development, and its inactivation can lead to developmental abnormalities and perinatal mortality in mice (Watzlawik et al., 2024). Therefore, *Pink1*’s role in neuronal health and its potential implications for Parkinson’s disease (PD) and aging underscore the need for further research to unravel its mechanisms and develop targeted therapies.

#### 5.2.3 Age-dependent decline in *Pink1* activity

The age-dependent decline in *Pink1* activity contributes significantly to mitochondrial dysfunction and neurodegeneration through a multifaceted mechanism involving the accumulation of mitochondrial damage, reduced expression, and impaired post-translational modifications. As individuals age, *Pink1*’s kinase activity is compromised by the loss of critical phosphorylation sites, which are essential for its catalytic function and ability to recruit Parkin for mitophagy (Yang et al., 2024). This decline leads to inefficient clearance of damaged mitochondria, exacerbating oxidative stress, calcium dysregulation, and the accumulation of toxic protein aggregates (e.g., *α*-synuclein), particularly in dopaminergic neurons. Additionally, *Pink1*’s broader roles in regulating mitochondrial calcium homeostasis, apoptosis, and transport are disrupted, further compromising neuronal health (Han et al., 2024). These age-related changes in *Pink1* activity not only correlate with increased vulnerability to neurodegenerative diseases like Parkinson’s disease but also present opportunities for therapeutic interventions targeting mitochondrial quality control pathways, such as enhancing *Pink1* function or upregulating compensatory mitophagy regulators like Nrf2, to potentially slow age-related neurodegeneration (Jia et al., 2025).

#### 5.2.4 *Pink1* and cellular senescence

*Pink1* plays a pivotal role in cellular senescence by orchestrating mitochondrial quality control and mitophagy, processes critical for determining cellular fate during aging. As a sensor of mitochondrial stress, *Pink1* accumulates on depolarized mitochondria, recruiting Parkin to initiate mitophagy—a mechanism that selectively removes damaged organelles to prevent the release of pro-senescent factors like reactive oxygen species (ROS) and mitochondrial DNA (mtDNA) fragments. This function is particularly vital in aged tissues, where mitochondrial damage accumulates due to reduced *Pink1* expression and activity, exacerbating oxidative stress and triggering senescence pathways (Zhu et al., 2025).

Dysfunctional mitochondria, exacerbated by *Pink1* deficiency, drive the activation of the senescence-associated secretory phenotype (SASP), a hallmark of senescent cells characterized by the secretion of inflammatory cytokines and chemokines. These SASP factors propagate senescence in neighboring cells and fuel chronic inflammation, further accelerating tissue aging. Moreover, *Pink1* interacts with key senescence regulators, such as p53/p21 and p16INK4a; its decline can lead to p53 activation and p21 upregulation, reinforcing cell cycle arrest and senescence (Shang et al., 2025).

In age-related diseases like PD and renal aging, *Pink1* dysfunction correlates with accelerated senescence. In Parkinson’s disease (PD), *Pink1* mutations impair mitophagy, causing dopaminergic neuron loss and senescence-like phenotypes, while in renal aging, *Pink1* deficiency activates the cGAS-STING pathway, driving inflammation and senescence (Zhou et al., 2025). Conversely, enhancing *Pink1* activity or mitophagy—via AMPK activation or Nrf2 upregulation—has shown promise in delaying senescence, as seen with drugs like metformin, which stimulate *Pink1*-dependent mitophagy to slow cellular aging. Thus, *Pink1* acts as a guardian of mitochondrial health, and its decline drives cellular senescence by promoting mitochondrial dysfunction, SASP, and interactions with senescence-regulating pathways. Targeting *Pink1*-mediated mitophagy may offer strategies to combat age-related decline and disease (Zhou et al., 2025).

#### 5.2.5 Comparative studies: *Pink1* in model organisms

Comparative studies across model organisms have significantly advanced understanding of *Pink1*’s role in cellular and organismal aging. In Drosophila, *Pink1*’s function in mitochondrial homeostasis and its links to PD have been extensively explored. Drosophila lacking *Pink1* exhibit reduced body weight, smaller cell sizes, and muscle degeneration, indicative of systemic growth inhibition and mitochondrial dysfunction. These phenotypes are rescued by human *Pink1* expression, underscoring functional conservation. Moreover, *Pink1* mutants display high blood glucose levels and insulin signaling suppression, likely due to increased secretion of the insulin antagonist Impl2. These findings suggest *Pink1*’s involvement in metabolic regulation beyond mitochondrial quality control (Bian et al., 2025).

In mice, *Pink1*’s role in neuroprotection has been clarified through studies contrasting its expression and function across species. While *Pink1* is nearly absent in mouse brains, it is highly expressed in human and monkey brains, where it acts as a cytosolic kinase rather than a mitochondrial protein. This species-specific expression pattern explains why *Pink1* knockout mice do not recapitulate PD-like neurodegeneration, unlike monkeys or humans. In mice, *Pink1*’s role in mitochondrial fission and synaptic plasticity has been highlighted. *Pink1*-mediated phosphorylation of Drp1 at Serine 616 (Drp1S616) promotes mitochondrial fission, essential for synaptic development and function. Loss of *Pink1* in mice leads to reduced dendritic spine maturation, axonal synaptic vesicle loss, and impaired long-term potentiation (LTP), all of which are rescued by expressing a phosphomimetic Drp1S616D mutant (Chen et al., 2025).

These studies collectively reveal *Pink1*’s dual role in mitochondrial dynamics and metabolic regulation, with species-specific variations in expression and function. While Drosophila models emphasize *Pink1*’s impact on growth and metabolism, mammalian models highlight its neuroprotective and synaptic functions, providing a comprehensive framework for understanding *Pink1*’s role in aging and disease.

*Pink1* (PTEN-induced kinase1) plays a pivotal role in the regulation of mitochondrial health and function, which are crucial for maintaining cardiovascular homeostasis. *Pink1* promotes mitophagy, the selective degradation of damaged mitochondria, thereby safeguarding cardiac cells from oxidative stress and promoting cardiac health (Guan et al., 2023). In mice models, deficiency of *Pink1* leads to mitochondrial dysfunction, increased oxidative stress, and impaired cardiac contractility (Paul and Pickrell, 2021). The morphology of the heart in animals with *Pink1* mutations often exhibits signs of cardiomyopathy, including enlarged hearts and reduced contractility (Yang et al., 2024). The incidence of heart dysfunction and arrhythmias in aging animals with compromised *Pink1* function increases significantly with age (Li et al., 2020). Studies in Drosophila have demonstrated that reduced *Pink1* activity is associated with a decline in cardiac performance and shortened lifespan (Imai, 2020). It is evident that *Pink1* plays a fundamental physiological role in the heart, being intimately linked to the maintenance of mitochondrial integrity and cardiac function. Mutations in the *Pink1* gene are known to cause early-onset Parkinson’s disease (PD), but their impact on cardiac aging is also increasingly recognized. These mutations disrupt the normal mitophagy process, leading to the accumulation of dysfunctional mitochondria and subsequent oxidative stress, which accelerates cardiac aging (Shin and Chung, 2022).

The etiology of PD associated with *Pink1* mutations is primarily genetic, and these mutations are believed to contribute to the development of PD and its cardiac comorbidities. *Pink1* deficiency can lead to a significant decrease in cardiac efficiency and an increased susceptibility to heart failure (Tan et al., 2021). Research has uncovered a strong correlation between PD and cardiovascular disease, with *Pink1* mutations potentially contributing to this link (Ke et al., 2024). Moreover, studies have shown that *Pink1* knockout or downregulation can exacerbate cardiac ischemia–reperfusion injury, highlighting its protective role against cardiac stress (Ma et al., 2023). Conversely, restoring *Pink1* function or enhancing mitophagy through pharmacological means has been shown to improve cardiac function and attenuate aging-related cardiac decline (Folkerts et al., 2023).

These findings suggest that mutations or dysfunction of the *Pink1* gene lead to abnormal mitochondrial homeostasis, cardiac dysfunction, and accelerated cardiac aging. Thus, *Pink1* emerges as a key regulator of cardiac health and aging, with potential therapeutic implications for the prevention and treatment of cardiac diseases associated with mitochondrial dysfunction.

#### 5.2.6 Impact of *Pink1* on myocardial mitochondrial dynamics

*Pink1* plays a critical role in regulating myocardial mitochondrial dynamics, particularly through its involvement in mitochondrial quality control and mitophagy. As a sensor of mitochondrial stress, *Pink1* accumulates on the outer mitochondrial membrane (OMM) of damaged mitochondria, where it recruits Parkin, an E3 ubiquitin ligase, to initiate mitophagy (Wang et al., 2024). This process selectively removes dysfunctional mitochondria, preserving the integrity of the mitochondrial network and preventing the release of pro-apoptotic factors.

In the myocardium, *Pink1*-mediated mitophagy is essential for maintaining cardiac function under stress conditions, such as ischemia–reperfusion injury (MIRI). Studies have shown that *Pink1* deficiency exacerbates mitochondrial fragmentation and cardiomyocyte apoptosis during MIRI, leading to impaired cardiac recovery. Conversely, enhancing *Pink1* activity, either genetically or pharmacologically, promotes mitophagy and reduces myocardial injury. For instance, overexpression of *Pink1* in mice protects against MIRI by accelerating the clearance of damaged mitochondria, while inhibition of *Pink1* exacerbates injury.

Additionally, *Pink1* interacts with other mitochondrial regulatory proteins, such as Drp1 (dynamin-related protein 1), to modulate mitochondrial fission and fusion. *Pink1*-mediated phosphorylation of Drp1 at Serine 616 promotes mitochondrial fission, which is crucial for mitochondrial quality control. Dysregulation of this pathway, such as through Drp1 inhibition, can lead to impaired mitophagy and exacerbated myocardial injury (Feng et al., 2024). Moreover, *Pink1*’s role extends beyond mitophagy to include regulation of mitochondrial biogenesis and energy metabolism. For example, *Pink1* interacts with transcription factors like PGC-1α to modulate mitochondrial gene expression, ensuring proper mitochondrial function and energy production (Zhao et al., 2024).

In summary, *Pink1* is a key regulator of myocardial mitochondrial dynamics, maintaining mitochondrial homeostasis through mitophagy, fission, and fusion. Its dysfunction contributes to mitochondrial pathology and cardiac dysfunction, making it a promising therapeutic target for heart diseases.

#### 5.2.7 *Pink1* deficiency and heart failure risk

*Pink1* deficiency has emerged as a pivotal contributor to heart failure (HF) risk by disrupting mitochondrial quality control and cardiomyocyte homeostasis. As a central regulator of mitophagy, *Pink1* ensures the selective removal of dysfunctional mitochondria, a process critical for maintaining mitochondrial integrity and energy production in the energy-demanding myocardium. Its deficiency leads to mitochondrial accumulation, oxidative stress, and cardiomyocyte apoptosis, manifesting as mitochondrial fragmentation, sarcoplasmic reticulum stress, and interstitial fibrosis—hallmarks of HF pathology. *Pink1*-null mice exhibit cardiomyopathy with reduced ejection fraction and exacerbated injury in ischemia–reperfusion and pressure-overload models, underscoring its role in cardiac vulnerability (Yang et al., 2024). Clinically, while direct links between *Pink1* variants and HF remain limited, patients with *Pink1* mutations may harbor subclinical cardiac abnormalities. Restoring *Pink1* function or enhancing downstream mitophagy pathways, such as through Parkin activation or antioxidative defenses, presents a promising therapeutic strategy to mitigate HF progression by addressing mitochondrial dysfunction at its core.

### 5.3 Exercise: the potent activator of *Pink1*

Emerging research has increasingly demonstrated that exercise plays a crucial role in enhancing mitochondrial function through the upregulation of *Pink1*, a critical regulator of mitophagy (Kang et al., 2025). Regular physical activity, particularly sustained aerobic exercise such as running, cycling, or swimming, has been shown to significantly elevate *Pink1* expression. This upregulation facilitates the selective removal of damaged or dysfunctional mitochondria via mitophagy, thereby reducing oxidative stress and improving mitochondrial quality control. By promoting the clearance of impaired organelles, exercise supports mitochondrial biogenesis—the generation of new, healthy mitochondria—and enhances overall cellular energy metabolism. These adaptive mechanisms are particularly relevant in the context of neurodegenerative disorders, where *Pink1* dysfunction is implicated in mitochondrial impairment and neuronal degeneration (Alghusen et al., 2024). Notably, animal studies have provided compelling evidence that exercise can partially compensate for mitochondrial deficits caused by *Pink1* deficiency. For instance, rodent models of PD with *Pink1* mutations exhibit improved mitochondrial function and reduced neurodegeneration following structured exercise regimens. These findings suggest that physical activity may serve as a viable non-pharmacological strategy to mitigate disease progression in PD and other conditions associated with *Pink1*-related mitochondrial dysfunction. The neuroprotective effects of exercise are thought to arise from enhanced mitophagy, reduced oxidative damage, and improved bioenergetic efficiency, collectively preserving neuronal health (Zhao et al., 2024).

Despite these promising observations, key questions remain regarding the optimal exercise parameters for maximizing *Pink1* activation. Current evidence does not yet establish whether resistance training, high-intensity interval training (HIIT), or moderate continuous aerobic exercise is most effective in upregulating *Pink1*-dependent pathways (Narendra and Youle, 2024). Additionally, the duration and frequency of exercise required to sustain long-term neuroprotective benefits warrant further investigation. Future research should also explore whether combining exercise with other mitochondrial-enhancing interventions, such as dietary modifications or pharmacological agents, could yield synergistic effects.

In summary, exercise represents a powerful, accessible intervention for enhancing *Pink1*-mediated mitophagy and mitochondrial health. Its potential to counteract neurodegeneration highlights the importance of incorporating physical activity into preventive and therapeutic strategies for Parkinson’s disease and related disorders. However, refining exercise protocols to optimize *Pink1* activation will be essential for translating these findings into clinical applications.

#### 5.3.1 Signal mechanisms of upregulation of *Pink1*/Parkin pathway by exercise (AMPK, SIRT1, BDNF/TrkB, etc.)

Exercise activates a convergent network of evolutionarily conserved signaling pathways that culminate in the enhanced expression and activity of *Pink1* and Parkin. A primary trigger is the increase in the cellular AMP/ATP ratio during exercise, which activates AMP-activated protein kinase (AMPK). AMPK acts as a master energy sensor and directly promotes mitophagy by phosphorylating ULK1, a key initiator of autophagy. Furthermore, AMPK activation can upregulate *Pink1* expression and facilitate the recruitment of Parkin to mitochondria. Concurrently, exercise elevates intracellular NAD^+^ levels, activating the deacetylase Sirtuin 1 (SIRT1). SIRT1 deacetylates transcription factors like FOXO3 and PGC-1α, promoting the expression of genes involved in mitochondrial biogenesis and quality control, including those in the *Pink1*/Parkin axis. Another critical exercise-induced factor is Brain-Derived Neurotrophic Factor (BDNF), whose levels rise in response to physical activity, especially aerobic exercise. BDNF signaling through its receptor TrkB activates downstream pathways such as PI3K/Akt and MAPK/ERK, which are known to support neuronal survival, synaptic plasticity, and mitochondrial health. This signaling cascade may indirectly stabilize *Pink1* function and enhance the cell’s capacity for stress-induced mitophagy. Together, AMPK, SIRT1, and BDNF/TrkB pathways form an integrated signaling network that sensitizes and strengthens the *Pink1*/Parkin-mediated mitochondrial clearance mechanism in response to exercise.

#### 5.3.2 Exercise-induced mitochondrial biosynthesis (PGC1α) and *Pink1*-mediated quality control synergize

The potent neuroprotective effect of exercise stems from its bidirectional optimization of the mitochondrial lifecycle: it simultaneously increases the population of healthy mitochondria by inducing biogenesis and removes dysfunctional ones by enhancing quality control mechanisms. These two processes do not operate in isolation; rather, they function synergistically through the coordinated actions of peroxisome proliferator-activated receptor gamma coactivator 1-alpha (PGC-1α) and the *Pink1*/Parkin pathway, collectively enabling the “renewal” and qualitative improvement of the mitochondrial network. Exercise, particularly aerobic exercise, serves as one of the most effective physiological activators of PGC-1α. Exercise-induced changes in cellular energy status (e.g., increased AMP/ATP ratio) and calcium signaling activate a cascade of kinases (such as AMPK and p38 MAPK), which directly phosphorylate and activate PGC-1α. Activated PGC-1α translocates to the nucleus, where it acts as a transcriptional coactivator. It binds to various transcription factors (e.g., nuclear respiratory factors NRF-1 and NRF-2), driving the robust expression of genes encoding mitochondrial electron transport chain components, antioxidant enzymes, and key factors for mitochondrial DNA replication (such as mitochondrial transcription factor A, TFAM). This ultimately leads to the generation of new, functionally intact mitochondria—a process defined as mitochondrial biogenesis.

Concurrently, exercise enhances *Pink1*/Parkin-mediated mitophagy through the aforementioned signaling pathways involving AMPK and SIRT1. This pathway is responsible for the precise recognition, tagging, and clearance of mitochondria that have become depolarized due to aging or damage. The essence of their synergy lies in establishing an efficient “replacement of the old with the new” cycle: PGC-1α-driven biogenesis provides a fresh supply of healthy mitochondrial “reserves,” ensuring that cellular energy supply is maintained without interruption after the removal of old organelles, thereby preserving metabolic homeostasis. P*ink1*/Parkin-mediated mitophagy ensures that defective mitochondria are promptly eliminated, preventing them from becoming persistent sources of reactive oxygen species (ROS) and releasing pro-apoptotic factors. This creates a “cleaner” cellular environment for the newly synthesized mitochondria.

This clearance function may itself, through signaling feedback, indirectly support the biogenesis process, forming a virtuous cycle. This synergy is particularly crucial in the context of Parkinson’s disease. Dopaminergic neurons are highly dependent on mitochondrial energy metabolism, and their mitochondrial networks are exceptionally vulnerable to damage. By simultaneously initiating both “construction” (biogenesis) and “cleanup” (mitophagy), exercise not only increases the absolute number of healthy mitochondria but, more importantly, significantly enhances the average functional quality of the entire mitochondrial population. This overall optimization of the mitochondrial network strengthens the neuron’s resilience against oxidative stress, protein misfolding, and energy crises, representing a core cellular mechanism through which exercise slows the progression of Parkinson’s disease. Therefore, viewing exercise as an integrative intervention that concurrently regulates both the “quantity” and “quality” of mitochondria provides a key perspective for understanding its disease-modifying potential.

#### 5.3.3 Comparative study of different types of exercise (aerobic, resistance, HIIT) on *Pink1*

Emerging evidence suggests that different exercise modalities may influence the *Pink1* pathway and mitochondrial health with varying efficacy, though the field requires further systematic comparison. Aerobic Exercise (e.g., running, cycling, swimming): This modality has the most substantial evidence base for activating the AMPK/SIRT1/PGC-1α axis and enhancing mitochondrial biogenesis and turnover. Studies in rodents and humans indicate that sustained aerobic activity is particularly effective in upregulating pathways linked to *Pink1* expression and mitophagy, making it a cornerstone for neuroprotective exercise prescriptions in PD. Resistance Training (e.g., weight lifting): While excellent for improving muscle mass, strength, and functional mobility, its direct impact on central nervous system *Pink1* signaling is less clearly defined. Benefits for PD patients may arise more from systemic metabolic improvements, increased IGF-1 levels, and enhanced neurotrophic support, which could indirectly support mitochondrial health. More research is needed to elucidate its direct effects on neuronal mitophagy.

High-Intensity Interval Training (HIIT): HIIT involves short bursts of maximal effort followed by recovery periods. It is highly efficient at improving cardiovascular fitness and metabolic parameters and has been shown to activate AMPK and PGC-1α powerfully. Preliminary data suggest it could potently stimulate mitochondrial adaptations. However, its feasibility, safety, and specific effects on the vulnerable dopaminergic system in PD patients require careful investigation in controlled clinical trials.

### 5.4 *Pink1* and high-fat diet (HFD) dysregulation

**Figure 5:**
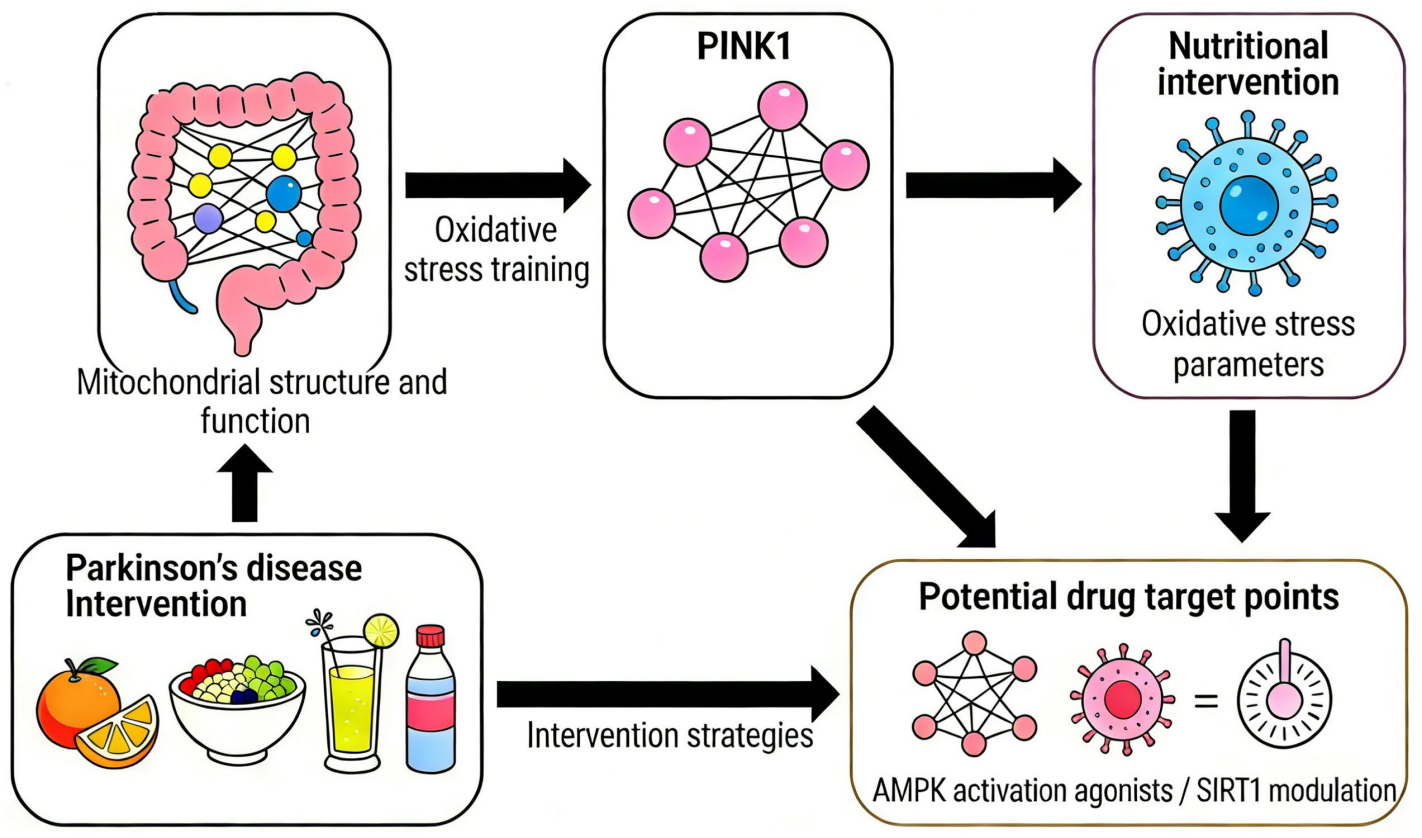
Depicts a *P*INK*1*-centered regulatory framework encompassing multi-step processes relevant to PD intervention. Initially, *P*INK*1* is localized to the intestinal structure (denoted as “Moting precture”); mediated by “Oxisance training,” this *P*INK*1* pool transitions to the Oxda/Atross signaling network—a process facilitated by Oxda/Atross training. This network establishes a functional connection with nutritional intervention (operationalized as “Mudinessam digay/groten display”) via Oxda/Atross-related parameters. Concomitantly, PD intervention strategies (including nutritional modalities) modulate this regulatory cascade, ultimately culminating in the identification of potential therapeutic targets: agonists targeting AMPK activation and modulators of SIRT1 activity.

In stark contrast to the beneficial effects of exercise, chronic consumption of a HFD, particularly one rich in saturated fats, acts as a potent metabolic stressor that disrupts cellular homeostasis. A key node of this disruption is the *Pink1*-mediated mitochondrial quality control system. HFD exposure initiates a cascade of metabolic disturbances that converge to impair *Pink1* expression, stability, and function, thereby crippling the cell’s primary defense against mitochondrial damage. This dietary-induced compromise of *Pink1* pathway integrity establishes a critical link between Western dietary patterns, accelerated mitochondrial decay, and increased vulnerability to Parkinson’s disease pathology (Figure 5).

#### 5.4.1 High fat diet (HFD): the metabolic disruptor of *Pink1*

A high-fat diet functions as a broad-spectrum metabolic disruptor, creating a cellular environment fundamentally at odds with optimal *Pink1* function. Unlike the transient, adaptive stress induced by exercise, HFD imposes a chronic state of metabolic overload. The excessive influx of lipids, especially saturated fatty acids like palmitate, overwhelms normal mitochondrial β-oxidation capacity and disrupts lipid metabolism. This leads to the pathological accumulation of lipid intermediates in non-adipose tissues, including neurons—a condition known as lipotoxicity. The resulting cellular milieu—characterized by lipid overload, insulin desensitization, and chronic low-grade inflammation—directly antagonizes the signaling pathways and energetic conditions required for efficient *Pink1*/Parkin pathway activation and mitophagy. Thus, HFD reconfigures the metabolic landscape in a way that suppresses the very mechanisms designed to maintain mitochondrial integrity.

#### 5.4.2 HFD inhibits *Pink1* function (lipotoxicity, insulin resistance, oxidative stress)

The inhibition of *Pink1* function by HFD is mediated through three interconnected mechanistic pillars:

Lipotoxicity: The defining feature of HFD, lipotoxicity, impairs *Pink1* through direct and indirect means. Excess saturated fatty acids can incorporate into mitochondrial membranes, disrupting their fluidity and integrity, which may interfere with the proper stabilization and accumulation of *Pink1* on the outer membrane following depolarization. Furthermore, lipid derivatives such as diacylglycerols and ceramides can activate serine/threonine kinases like Protein Kinase C (PKC) and c-Jun N-terminal kinase (JNK). These kinases can phosphorylate components of the mitophagy machinery, potentially disrupting the recruitment or activity of Parkin. Lipotoxicity also promotes endoplasmic reticulum (ER) stress, which activates the unfolded protein response (UPR). A chronically activated UPR can divert cellular resources, alter global protein synthesis, and create cross-talk that negatively impacts mitochondrial autophagy pathways.

Insulin Resistance: HFD is a primary driver of systemic and central nervous system insulin resistance. Insulin signaling, via the PI3K/Akt pathway, is a crucial pro-survival signal that supports mitochondrial function and biogenesis. When this pathway is blunted, its supportive role for cellular homeostasis is diminished. Insulin resistance can lead to reduced activation of transcription factors like FOXO3, which are involved in stress resistance and autophagy regulation. Moreover, impaired neuronal insulin signaling alters glucose metabolism and energy sensing, which may indirectly affect the AMPK activity that is vital for initiating mitophagy. This loss of insulin’s trophic support makes cells less capable of mounting an effective *Pink1*-mediated response to mitochondrial damage.

Oxidative Stress: HFD-induced mitochondrial overload and dysfunction inevitably increase the production of reactive oxygen species (ROS). While moderate ROS can act as signaling molecules, the chronic, excessive oxidative stress from HFD has deleterious effects. High levels of ROS can cause direct oxidative damage to the *Pink1* protein itself, potentially inactivating its kinase domain. Oxidative stress also promotes the oxidation of Parkin and ubiquitin, which can inhibit the ubiquitin ligase activity of Parkin and the formation of the phosphorylated ubiquitin chains essential for mitophagy progression. This creates a vicious cycle: mitochondrial dysfunction generates ROS that disable the *Pink1*/Parkin system, leading to further accumulation of dysfunctional, ROS-producing mitochondria.

#### 5.4.3 HFD-induced neuroinflammation and microglia activation interfere with *Pink1* pathway

In contrast to the beneficial effects of exercise, a HFD has been shown to disrupt *Pink1*-mediated mitochondrial quality control, contributing to metabolic dysfunction and neurodegeneration. Chronic consumption of a HFD leads to mitochondrial stress and oxidative damage, yet paradoxically impairs *Pink1*-dependent mitophagy, resulting in the accumulation of dysfunctional mitochondria. This dysregulation is further exacerbated in the context of obesity and insulin resistance, where decreased *Pink1* activity has been observed. Animal models of Parkinson’s disease (PD) demonstrate that a HFD exacerbates *Pink1* deficiency-related neurodegeneration, suggesting a critical link between dietary lipids and mitochondrial homeostasis. Therapeutic strategies, such as dietary modification (e.g., ketogenic or Mediterranean diets) or pharmacological *Pink1* activation, may help counteract these detrimental effects and restore proper mitochondrial function (Martínez-González and Hernández Hernández, 2024). Further research is needed to elucidate the precise mechanisms by which lipid metabolism influences *Pink1* signaling and to develop targeted interventions for metabolic and neurodegenerative disorders.

**Figure 6:**
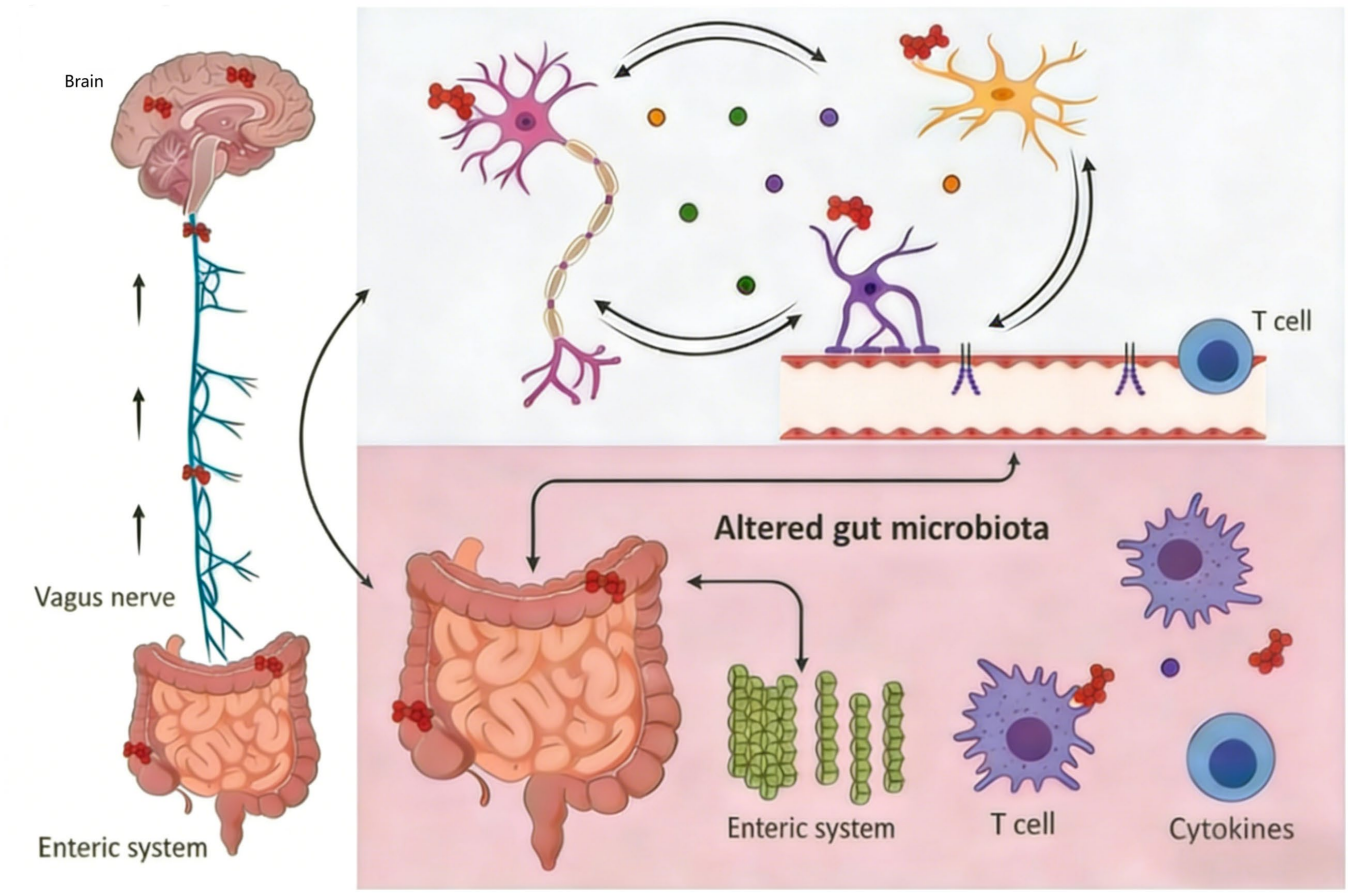
Elaborates on the intricate gut-brain axis interaction: It begins with altered gut microbiota—dysregulated microbial communities in the enteric system that reshape local immune activity. These microbiota signal to gut-resident immune cells (including macrophages and T cells), prompting them to secrete pro-inflammatory or regulatory cytokines. These cytokines then transmit signals along the vagus nerve—the primary neural link between the enteric system and the brain—facilitating communication across the gut-brain barrier. Upon reaching the central nervous system, these signals target key brain cells: microglia (the brain’s immune cells), neurons (which mediate neural activity), and astrocytes (support cells that regulate brain homeostasis). Additionally, brain-resident cells (such as brain-infiltrating T cells) engage in reciprocal cross-talk with these targeted cells, creating a bidirectional loop. This entire pathway bridges functional changes in the enteric system (driven by microbiota shifts) to dynamic regulation of the central nervous system, highlighting the interconnectedness of gut and brain physiology.

Emerging research indicates that chronic HFD consumption significantly impairs *Pink1*-regulated mitochondrial homeostasis, creating a detrimental cycle of metabolic and neurological dysfunction. The excessive lipid load from HFD induces mitochondrial oxidative stress while simultaneously suppressing *Pink1*-mediated mitophagy, leading to dangerous accumulation of damaged mitochondria. This disruption is particularly pronounced in states of metabolic syndrome, where obesity and insulin resistance correlate with marked reductions in *Pink1* activity. Notably, preclinical studies reveal that HFD exacerbates neurodegeneration in *Pink1*-deficient models, accelerating both motor and cognitive decline. The mechanisms may involve lipid-induced modifications to *Pink1*’s kinase activity or its interaction with downstream effectors like Parkin (Guo et al., 2024). While dietary interventions such as Mediterranean or time-restricted feeding regimens show promise in restoring *Pink1* function, the development of small-molecule *Pink1* activators may offer more targeted therapeutic potential. Current research gaps include understanding tissue-specific effects of HFD on *Pink1* and determining whether early dietary interventions can prevent *Pink1* dysregulation in at-risk populations. These findings underscore the critical interplay between nutrition and neuronal survival pathways, suggesting that dietary management could be a viable strategy for mitigating *Pink1*-related disorders (Figure 6).

## 6 Interplay and therapeutic implications: an integrated perspective

The preceding sections have established *Pink1* as a central molecular switch, its function delicately balanced by the opposing forces of aging, exercise, and diet. Understanding the dynamic interplay among these three factors is crucial for developing a holistic view of PD pathogenesis and for designing effective, personalized intervention strategies. This integrated perspective moves beyond viewing each factor in isolation, instead examining how they converge on the *Pink1* pathway to ultimately determine neuronal fate, and what therapeutic opportunities this convergence presents.

### 6.1 Crossing effects of aging, exercise and high-fat diet on *Pink1* pathway

The influences of aging, exercise, and HFD on the *Pink1* pathway are not merely additive; they interact in complex, often antagonistic ways, creating a net effect on mitochondrial health and PD risk. Aging and HFD: A Synergistic Downward Spiral. Aging and HFD likely exert additive or synergistic negative effects. Both independently suppress *Pink1* function—aging through transcriptional decline and proteostatic collapse, and HFD through lipotoxicity and metabolic disruption. When combined, they can create a vicious cycle: age-related decline in autophagy makes cells less capable of handling HFD-induced lipid overload and mitochondrial damage, while HFD-induced inflammation and oxidative stress accelerate cellular aging processes, including further suppression of *Pink1*. This synergy explains the exponentially increased risk of age-related neurodegenerative diseases in populations with long-term unhealthy dietary habits.

Exercise as a Counteracting Force. Exercise serves as a powerful antagonist to both aging- and HFD-induced *Pink1* suppression. It can directly counteract the molecular hallmarks of aging (e.g., by activating AMPK/SIRT1 to improve energy sensing and stress resistance) and mitigate the metabolic disturbances of HFD (e.g., by improving insulin sensitivity and reducing systemic inflammation). By potently upregulating the *Pink1* pathway, exercise can “rescue” impaired mitophagy and boost mitochondrial biogenesis, thereby breaking the vicious cycle promoted by aging and poor diet. The neuroprotective benefit of exercise is thus most pronounced in contexts of high risk (aging) or metabolic challenge (HFD).

The Complex Role of Dietary Fat: HFD vs. Ketogenic Diet (KD). This interplay further necessitates distinguishing between different high-fat dietary patterns. While a typical Western-style HFD is detrimental, a well-formulated KD may have a divergent effect. KD, by inducing nutritional ketosis, can activate protective pathways (e.g., via β-hydroxybutyrate-mediated inhibition of HDACs and activation of Nrf2) that may support mitochondrial function and confer neuroprotection in some models, potentially modulating the negative impact of aging or even mimicking certain beneficial effects of exercise on cellular metabolism. This highlights that the “quality” and metabolic context of dietary fat are critical determinants of its ultimate effect on the *Pink1* axis.

### 6.2 Potential intervention strategies based on *Pink1* regulation: exercise prescription and nutritional supplementation

The centrality of the *Pink1* pathway in integrating lifestyle signals makes it a prime target for developing non-pharmacological and pharmacological interventions aimed at PD prevention and modification. Precision Exercise Prescription: Moving beyond generic advice, future strategies should involve personalized exercise regimens designed to optimally stimulate the *Pink1* pathway. This includes determining the most effective modality (likely aerobic exercise as a foundation), optimal intensity, duration, and frequency for different stages of PD or levels of risk. Combining aerobic exercise with resistance training may offer comprehensive benefits for both neuronal and systemic health. The concept of “exercise mimetics”—drugs that activate the same pathways as exercise (e.g., AMPK activators)—is an active area of research for those unable to engage in physical activity. Targeted Nutritional Supplementation and Dietary Patterns: Nutrition strategies should focus on counteracting HFD-induced damage and supporting *Pink1* function. This involves: Promoting dietary patterns rich in polyphenols, antioxidants, and healthy fats (e.g., Mediterranean diet) to reduce oxidative stress and inflammation. Investigating specific supplements that may enhance mitophagy or mitochondrial biogenesis, such as compounds that activate AMPK (e.g., certain polyphenols like resveratrol) or Nrf2 (e.g., sulforaphane). Carefully exploring the therapeutic window and formulation for ketogenic diets in PD, understanding its potential benefits and risks in different patient subgroups.

Pharmacological *Pink1* Pathway Activators: The direct development of small-molecule *Pink1* kinase activators or Parkin function enhancers represents a promising frontier for drug discovery. These agents would aim to directly boost the pathway’s activity, bypassing upstream defects caused by aging or genetics. Additionally, compounds that enhance general autophagy or improve lysosomal function (e.g., TFEB activators) could synergize with lifestyle interventions to bolster the entire mitochondrial quality control system.

## 7 Conclusion

In this review, we have meticulously delved into various studies investigating the intricate interplay between the *Pink1* gene and PD, in the context of aging, exercise regimens, and dietary patterns, particularly HFD. Our analysis underscores that *Pink1*, a pivotal player in mitochondrial homeostasis, is intricately linked to the aging process and essential for maintaining neuronal health and function. Mutations or disruptions in *Pink1* can lead to profound consequences, including the onset and progression of PD, characterized by dopamine neuron degeneration and motor impairment. Notably, we found that engaging in regular exercise routines, coupled with a balanced diet, can mitigate the detrimental effects of Pink1 dysfunction-mediated PD, potentially preserving mitochondrial integrity and delaying neuronal decline.

However, the intricate relationship between exercise, a HFD, and Pink1 remains an understudied area that merits further exploration. Specifically, research should focus on elucidating whether tailored exercise programs can alleviate or reverse mitochondrial dysfunction and neuronal loss caused by *Pink1* mutations or deficiencies, a facet that has been sparsely investigated thus far. Additionally, the interplay between a HFD and *Pink1* function in the context of PD pathogenesis requires deeper scrutiny, as dietary interventions may offer promising avenues for disease management.

By delving deeper into the intricate relationship between Pink1, exercise, diet, and PD, we aim to gain a more holistic understanding of the underlying mechanisms and devise more effective strategies for the prevention and treatment of this devastating neurodegenerative disorder. Beyond lifestyle interventions, direct pharmacological targeting of the PINK1/Parkin pathway has emerged as a promising frontier for disease-modifying therapy in PD. Small-molecule PINK1 activators (e.g., kinetin derivatives) and Parkin activators have demonstrated efficacy in preclinical models, while gene therapy approaches using AAV-mediated PINK1 delivery or CRISPR-based base editing are advancing toward clinical translation; a Phase I trial of kinetin triphosphate (KTP) in PINK1-related PD was initiated in late 2025, and a first-in-human AAV9-PINK1 gene therapy trial is currently recruiting patients with biallelic PINK1 mutations. Importantly, emerging evidence suggests that sex and population differences may influence PINK1 function and therapeutic responsiveness—female Pink1 knockout mice exhibit less severe motor deficits than males, and PINK1 mutation frequency varies across ethnic groups—yet these dimensions remain critically understudied, underscoring the need for diverse cohort recruitment and sex-stratified analyses in future studies.

Several critical knowledge gaps warrant further investigation. First, the optimal exercise parameters (modality, intensity, duration) for maximizing PINK1-mediated mitophagy remain undefined, necessitating randomized controlled trials with biomarker endpoints. Second, a fundamental distinction exists between monogenic PINK1 deficiency (early-onset PD) and age-related functional decline (sporadic PD), which may require distinct therapeutic strategies. Third, the tissue-specific and cell-type-specific roles of PINK1—particularly in peripheral tissues such as gut epithelium and immune cells—and their contribution to systemic PD pathology remain poorly understood. Fourth, significant species differences (e.g., *Pink1* knockout mice lack robust dopaminergic neuron loss) highlight the need for humanized models and non-human primate studies to ensure translational relevance. Addressing these gaps will be essential for translating mechanistic insights into effective, personalized interventions for PINK1-related PD.

## 8 Knowledge gaps and future directions

Despite substantial progress in understanding PINK1 biology and its modulation by lifestyle factors, several critical knowledge gaps remain. Addressing these gaps will be essential for translating mechanistic insights into clinical benefit.

### 8.1 Exercise prescription and mechanistic heterogeneity

Exercise unequivocally benefits PD patients, but the optimal modality, intensity, duration, and frequency for maximizing PINK1-mediated mitophagy remain undefined. Aerobic exercise robustly activates AMPK/SIRT1/PGC-1α signaling, yet whether resistance training or high-intensity interval training (HIIT) confers comparable or synergistic effects on neuronal PINK1 is unclear. Future studies should employ randomized controlled trials with biomarker endpoints—such as PINK1 activity in peripheral blood mononuclear cells—to establish evidence-based exercise prescriptions.

Beyond optimizing exercise parameters, a deeper mechanistic understanding of how exercise engages the PINK1 pathway is essential for developing targeted interventions. In this context, a critical distinction must be drawn between monogenic PINK1 deficiency—which causes early-onset autosomal recessive PD through complete loss of kinase function—and age-related PINK1 dysfunction, characterized by progressive downregulation, impaired post-translational activation, or defective mitochondrial translocation without genetic mutation. While both converge on impaired mitophagy and mitochondrial accumulation, their underlying molecular pathologies differ: genetic deficiency abolishes the initiating signal for Parkin recruitment, whereas age-related dysfunction often preserves residual PINK1 activity but disrupts its coupling to downstream effectors, potentially rendering it more amenable to pharmacological or lifestyle-based enhancement.

The beneficial effects of exercise on PINK1-mediated mitophagy are orchestrated through a hierarchically organized signaling network. At the apex, AMPK serves as the primary energy sensor, activated by exercise-induced increases in the AMP/ATP ratio. AMPK directly phosphorylates and activates ULK1 to initiate autophagy, while also upregulating Pink1 transcription through downstream transcription factors. Parallel to AMPK, SIRT1 is activated by exercise-induced NAD^+^ elevation and functions as a critical amplifier, deacetylating and activating PGC-1α and FOXO3 to promote mitochondrial biogenesis and stress resistance. PGC-1α operates at an intermediate level, integrating signals from both AMPK and SIRT1 to coordinate nuclear-encoded mitochondrial gene expression. BDNF, secreted in response to exercise, acts through its receptor TrkB to activate PI3K/Akt and MAPK/ERK cascades, providing trophic support that indirectly sustains PINK1 stability and autophagic capacity. Available evidence suggests a hierarchical relationship wherein AMPK activation represents the most immediate and non-redundant trigger for exercise-induced mitophagy, with SIRT1 and PGC-1α functioning as reinforcing modulators that amplify and sustain the response, while BDNF/TrkB signaling confers parallel neuroprotective effects that are partially independent but synergistic with the core AMPK-PINK1 axis. This hierarchical framework has direct implications for intervention design: strategies that fail to engage AMPK are unlikely to robustly activate PINK1-mediated mitophagy, whereas SIRT1 or PGC-1α modulators alone may yield partial benefits that require concurrent AMPK engagement for full efficacy.

A fundamental distinction also exists between monogenic PINK1 deficiency (early-onset, autosomal recessive PD) and age-related PINK1 functional decline (sporadic PD). These two contexts may require distinct therapeutic strategies. Whether PINK1 activators developed for genetic PD will benefit patients with age-related PINK1 downregulation remains unknown. Future research must clarify whether partial activation suffices in aging contexts versus complete restoration required in genetic cases. Additionally, PINK1 is expressed across multiple tissues, yet most studies focus on dopaminergic neurons. The peripheral roles of PINK1—in immune cells, gut epithelium, and cardiac tissue—and their contribution to systemic PD pathology are poorly understood. Given emerging evidence of gut-brain axis involvement, future studies should explore whether lifestyle interventions modulate PINK1 in peripheral tissues and whether this modulation contributes to neuroprotection.

### 8.2 Translational validity and population diversity

Significant species differences exist in PINK1 expression and function. Pink1 knockout mice do not recapitulate dopaminergic neuron loss seen in humans, whereas non-human primate models exhibit more faithful pathology. Developing humanized models—such as iPSC-derived neurons with patient-specific mutations and organoid systems—and validating findings in non-human primates will be critical for ensuring translational relevance.

Emerging evidence also suggests that PINK1 expression, function, and therapeutic responsiveness may vary across populations. Female Pink1 knockout mice exhibit less severe motor deficits than males, potentially due to estrogen-mediated compensation. PINK1 mutation frequency varies geographically, with higher prevalence in North African and Southeast Asian populations. Yet most studies have been conducted in European-derived cohorts. Future research must prioritize diverse cohort recruitment and sex-stratified analyses to ensure that emerging therapies are effective across all affected populations.
